# NCSTN promotes hepatocellular carcinoma cell growth and metastasis via β-catenin activation in a Notch1/AKT dependent manner

**DOI:** 10.1186/s13046-020-01638-3

**Published:** 2020-07-06

**Authors:** Hui Li, Tian Lan, Lin Xu, Hailing Liu, Jinju Wang, Jiaxin Li, Xiangzheng Chen, Jiwei Huang, Xuefeng Li, Kefei Yuan, Yong Zeng, Hong Wu

**Affiliations:** 1 Department of Liver Surgery, Liver Transplantation Division, Laboratory of Liver Surgery, West China Hospital, Sichuan University, Chengdu, 610041 China; 2 Laboratory of Liver Surgery, West China Hospital, Sichuan University, Chengdu, 610041 China; 3 School of Basic Medical Sciences, Guangzhou Medical University, Guangzhou, 511436 China; 4 Shenzhen Luohu People’s Hospital, The Third Affiliated Hospital of Shenzhen University, Shenzhen, 518001 China

**Keywords:** Hepatocellular carcinoma, γ-Secretase, Nicastrin, Epithelial–mesenchymal transition, β-Catenin

## Abstract

**Background:**

Hepatocellular carcinoma is the third top cause of cancer-related mortalities worldwide. The prognosis of HCC patients remains poor due to rapid progression and high incidence of tumor recurrence. Nicastrin (NCSTN), a core subunit of γ-Secretase, has been reported to play a vital role in tumor progression. However, no study till now has revealed its role in HCC.

**Methods:**

The expression of NCSTN was evaluated by immunohistochemical staining, Western blot, and quantitative real-time PCR. Cell counting kit-8, colony formation and cell cycle assays were used for evaluating cell growth in vitro. Transwell and wound-healing assays were used for evaluating cell migration and invasion capacity. Immunofluorescence, subcellular protein fractionation and co-immunoprecipitation were used for location analysis of β-catenin. The in vivo functions of NCSTN were illustrated by xenograft tumor models.

**Results:**

NCSTN was dramatically overexpressed in HCC compared to normal liver tissues. Elevated NCSTN expression level was significantly correlated to worse overall and recurrence-free survival of HCC patients. Enhanced NCSTN expression promoted HCC cell growth, migration and invasion in vitro and in vivo. Mechanistic investigations showed that NCSTN induced epithelial-mesenchymal transition (EMT) process via upregulation of Zeb1. Subsequently, we revealed that NCSTN facilitated nuclear translocation of β-catenin, a positive transcriptional regulator of Zeb1. Using Notch and AKT inhibitors, we revealed that NCSTN promoted β-catenin activation through Notch1 and AKT signaling pathway. NCSTN increased AKT and GSK-3β phosphorylation by cleavage of Notch1, which decreased GSK-3β/β-catenin complex. The inactivation of GSK-3β inhibited the β-catenin degradation and promoted nuclear translocation of β-catenin to initiate transcription of Zeb1, resulting in malignant phenotype.

**Conclusions:**

Our results demonstrated that NCSTN promoted HCC cell growth and metastasis via β-catenin-mediated upregulation of Zeb1 in a Notch1/AKT dependent manner, suggesting that NCSTN might serve as a potential prognostic marker and therapeutic target for HCC.

## Background

Hepatocellular carcinoma (HCC), one of the most commonly diagnosed human malignancies, is the third top cause of cancer-related mortalities worldwide [1]. Despite continuous advances in diagnosis and treatments over decades, the prognosis of HCC patients remains poor due to rapid progression and high incidence of tumor recurrence [2]. It is extremely urgent to illuminate the molecular mechanisms underlying HCC progression as well as identify effective therapeutic strategies against HCC progression.

γ-Secretase, a transmembranous multiprotein enzyme complex, consists of four core subunits: presenilin-1, nicastrin (NCSTN), anterior pharynxdefective phenotype-1 and the presenilin enhancer-2 [3]. The γ-Secretase complex functions as a protease to cleave and activate various transmembrane proteins [4]. Evidence is mounting that NCSTN is essential for the intracellular trafficking and stability of γ-Secretase, as well as recognition of γ-Secretase substrates [5]. As a transmembrane protein, NCSTN contains extracellular and transmembrane domains, which are identified to be the functional sites for recruitment of γ-Secretase substrates [6]. Previous studies have examined the upregulated expression level of NCSTN in breast cancer and demonstrated the oncogenic role of NCSTN in vivo and in vitro assays [7, 8]. NCSTN overexpression regulated the properties of breast cancer stem cells and induced epithelial-mesenchymal transition (EMT) via cleavage of Notch1 [8]. Besides, it has been revealed that NCSTN was correlative to response to chemotherapy for colon cancer [9]. Additionally, the specific monoclonal antibodies against NCSTN were evaluated in cellular and pre-clinical assays, which suggested a promising role of targeting NCSTN for the treatment of invasive triple negative breast cancer [10]. However, no study till now has examined the expression of NCSTN and its biological relevance in tumor progression in HCC.

In the present study, we found that NCSTN was up-regulated in HCC and could serve as an independent prognostic factor for HCC patients. By stable silencing and overexpression of NCSTN, we addressed the crucial effects of NCSTN on proliferative and invasive properties of HCC cells in vitro and vivo. We revealed that increased expression of NCSTN promoted the invasive capacity of HCC cells through activation of β-catenin, which subsequently induced EMT process. Further investigation demonstrated NCSTN-mediated intramembranous cleavage of Notch 1 and activation of AKT signaling as key regulators for malignant phenotype of HCC cells. Collectively, our findings indicate that NCSTN promotes HCC cell growth and metastasis through β-catenin activation in a Notch1/AKT dependent manner, and may be characterized as a promising target for HCC therapeutic strategies.

## Materials and methods

### Study population and specimens

A total of 245 samples were obtained from surgically treated HCC patients who had not receive preoperative treatments at West China Hospital (Chengdu, China) from 2009 to 2013. Of them, 108 samples contained HCC and paired adjacent tissues, were used for investigating the expression difference between tumor and adjacent tissues. The total 245 samples were used for quantification of NCSTN expression and analysis of their correlation with patient outcomes after hepatectomy. This study has been approved by the ethics committee of West China Hospital.

### Tissue microarray and immunohistochemistry (IHC)

The tissue microarrays and IHC analysis were performed as we previously described [11]. Two independent pathologists evaluated the staining. Quantitative analyses were performed by summing the staining intensity score (0, negative; 1, weak; 2, moderate; 3, strong) of ten randomly selected fields. The cut-off value for classification of patients was 150 points. The antibodies for IHC were shown in Table S1 (Additional file 1).

### Western blotting (WB)

Tissues and cells were lysed in RIPA buffer, which was supplemented with protease and phosphatase inhibitor (Thermo Fisher Scientific, CA, USA), and then quantified by using BCA protein assay kit (Beyotime Biotechnology, China). The WB assay was conducted as we previously described [12]. The intensity of signals was scanned using ChemiDoc MP Imager System (Bio-Rad, California, USA). The primary antibodies for WB were shown in Table S1 (Additional file 1).

### The Cancer genome atlas (TCGA) data analysis

High-throughput RNA-sequencing data of 371 samples and 50 matched adjacent samples were downloaded from the TCGA dataset. Of them, 7 were confirmed as HCC plus intrahepatic cholangiocarcinoma (ICC) by histology and were excluded. Therefore, 364 HCC patients were finally included in this study (Additional file 3). The software of X-tile (version 3.6.0, Yale University School of Medicine) was used for identification of optimal cut-off value for NCSTN based on the association between mRNA expression level and overall survival (OS).

### Cell culture and reagents

Human HCC cell lines Hep3B, Huh7, HepG2 and HCCLM3 were purchased from Cell Bank of Shanghai Institutes for Biological Science, Chinese Academy of Science (Shanghai, China), SNU449 and SNU387 were purchased from American Type Culture Collection (ATCC, Manassas, VA, USA). Cells were cultivated in recommended medium, supplemented with 10% FBS, 100 μg/mL streptomycin and 100 U/mL penicillin, at 37 °C with 5% CO2. The Notch inhibitor FLI-06 and AKT inhibitor MK-2206 2HCl were obtained from Selleck (Houston, TX, USA).

### Cell transfection

Lentiviral vector containing human NCSTN sequence and lentiviral particles containing shNCSTN-1 and shNCSTN-2 were synthesized by GenePharma (Shanghai, China). The specific small interfering RNA (siRNA) oligonucleotides targeting β-catenin and Zeb1 were synthesised by Viewsolid Biotech (Beijing, China). 1 × 10^6^ cells were seeded in 6-well plate and transfected with specific siRNA using GenmuteTM Reagent (SignaGen Laboratories, Maryland, USA) according to the manufacturer’s instructions, and then harvested 48 h post transfection. The sequences of siRNA and shRNA used in this study were showed in Table S3 (Additional file 1).

### In vitro cell proliferation and cell cycle assays

2 × 10^3^ cells within 100 μl medium were seeded in triplicate wells of 96-well plate and observed for 120 h. Each well was co-cultured with 10 μl Cell Counting Kit-8 (CCK-8) (Beyotime Biotechnology, Shanghai, China) and incubated at 37 °C for 2 h. The absorbance was detected at 450 nm by using the EonTM Microplate Reader (BioTek, VT, USA). 1 × 10^3^ cells were seeded in triplicate wells of 6-well plate and cultured for 2 weeks. After fixed with 4% paraformaldehyde for 20 min, the colonies were stained using 0.1% crystal violet and counted. According to the manufacturer’s instructions of Cell Cycle Kit (4A Biotech, Beijing, China), cells were fixed using 95% cold ethanol, followed by incubation with propidium iodide at 37 °C for 30 min and analyzed by the CytoFLEX Research Flow Cytometer (Beckman Coulter, CA, USA).

### In vitro wound-healing assay

Cells were plated in triplicate into 6-well plates. A standard 10 μl pipette tip was used to scratch wound when the cells reached a density of 95%. Subsequently, the cells were cultured in FBS-free medium. After 24 or 48 h, the wound closure was captured by a microscope and calculated using the software of Image J (National Institutes of Health, Bethesda, MD, USA).

### In vitro transwell migration and matrigel invasion assay

Transwell chambers (8.0 μm pore size, Corning Costar, Kennebunk, USA) were applied in migration and matrigel invasion assays. For migration assay, 3 × 10^4^ cells were suspended in FBS-free DMED medium and then seeded into upper chamber. For matrigel invasion assay, 5 × 10^4^ cells were suspended in FBS-free medium and seeded into upper chamber with matrigel coating. DMEM medium containing 10% FBS were added to the lower chamber. After cultivation for 24 or 48 h, cells which migrated or invaded onto the lower surface of lower chambers were fixed using 4% paraformaldehyde, followed by stained using 0.1% crystal violet. Cells of 5 randomly selected fields were calculated and counted using Image J.

### In vivo xenograft assay

The in vivo animal experiment was authorized by the Animal Ethic Review Committees of the West China Hospital. 5-week old male BALB/c nude mice were obtained from HFK BIOSCIENCE (Beijing, China) and used for in vivo assays. 1 × 10^6^ cells (HCCLM3) or 2 × 10^6^ cells (Hep3B) were suspended using 100 μl PBS and subsequently implanted subcutaneously into right axillas of mice (5 mice for each group). Measurement of tumor size was performed weekly by using the formula of length × width^2^ × 0.52. The mice were sacrificed at 4 or 6 weeks after implantation, followed by measure of tumor weight. For liver orthotopic-implanted HCC models, 1 × 10^6^ cells were implanted into left lobe after mice were anesthetized. Mice were sacrificed 4 weeks after implantation and the livers were scanned using the IVIS@ Lumina II system (Caliper Life Sciences, Hopkinton, MA, USA). 2 × 10^6^ cells were used for construction of lung metastasis models by injection into tail veins and the mice were sacrificed at the time of 6 weeks post implantation and the lungs were scanned using the IVIS@ Lumina II system. The metastatic foci were confirmed by hematoxylin and eosin staining.

### RNA extraction and quantitative real-time PCR

Total RNAs of HCC cells were extracted by using Cell Total RNA Isolation Kit (Foregene, Chengdu, China) in accordance with the manufacturer’s instructions. The first-strand complementary DNA was synthesized using HiScript II Reverse Transcriptase (Vazyme, Nanjing, China). ChamQ™ SYBR@ qPCR Master Mix (Vazyme Biotech, Nanjing, China) was utilized for real-time PCR. All the reactions were performed in triplicate and the relative mRNA expression levels were quantitated using the 2^-ΔΔ^CT method. U6 was utilized as endogenous control. Primers used in this study were summarized in Table S2 (Additional file 1).

### Immunofluorescence (IF) analysis

3 × 10^3^ cells were plated on cover slips in 24-well plates. The cells were fixed using 4% paraformaldehyde at room temperature for 20 min, premeabilized using 0.2% Triton X-100 in PBS for 10 min and then blocked with 5% BSA at room temperature for 1 h, followed by incubation of primary antibodies at 4 °C overnight. Then the cells were washed using PBST (0.1% Tween-20 in PBS) for tree times and incubated with appropriate fluorophore-conjugated secondary antibodies (1:1000, Thermo Fisher Scientific, CA, USA). After incubation with DAPI (Kaiji, Nanjing, China), the cover slips were mounted on slides and scanned using AX10 imager A2 microscope (Carl Zeiss MicroImaging).

### Subcellular protein fractionation

Cytoplasmic and nuclear protein fractions were extracted by using the NE-PER™ Nuclear and Cytoplasmic Extraction Reagents (Thermo Fisher Scientific, CA, USA) according to its protocol. The extracted protein samples were subsequently analyzed by WB assay. The β-Tubulin and Histone H3 were used as cytoplasmic and nuclear endogenous control, respectively.

### Luciferase reporter assay

For TOP/FOP Flash assay, 1 × 10^4^ cells were seeded in triplicate in 24-well plate 24 h prior to transfection. They were co-transfected with the pRL-CMV plasmid (Renilla luciferase, Promega) plus either TOP-Flash or FOP-Flash plasmid (Upstate) according to the instructions of Lipofectamine 3000 (Invitrogen, Carlsbad, CA, USA). Cells were cultured for 48 h and then the luciferase activity were evaluated using Duo-Luciferase HS Assay Kit (GeneCopoeia, CA, USA) in accordance with the manufacturer’s instructions. The results were shown as TOP/FOP Flash activity. For Zeb1 promoter activity luciferase reporter assay, pRP-Zeb1 were transfected into indicated cells. Firefly and renilla luciferase activities were detected after 48 h. The relative luciferase activity was shown as firefly/renilla luciferase activity.

### Chromatin immunoprecipitation assay

Indicated cells were cross-linked using 1% paraformaldehyde and quenched by glycine solution [13]. The chromatin immunoprecipitation (ChIP) assay was performed using the Pierce Magnetic ChIP Kit (Thermo Fisher Scientific), in accordance with the manufacturer’s instructions. Anti–β-catenin antibody and normal IgG (Millipore) were used for immunoprecipitation. ChIP-enriched DNA samples were quantified by real-time PCR to determine the special binding sites (BS) of the Zeb1 promoter region. The sequences of primers used for real-time PCR were summarized in Table S2 (Additional file 1).

### Co-immunoprecipitation (co-IP)

Co-IP was conducted using Pierce Crosslink Magnetic IP/Co-IP Kit in accordance with the manufacturer’s instructions (Thermo Fisher Scientific, CA, USA). Briefly, cell lysates were incubated with protein A/G magnetic beads which were previously bind to primary antibodies, then dissociated the bound antigens from antibody-crosslinked beads by eluting in a low-pH elution buffer. The eluent was detected by WB analysis.

### Statistical analysis

All the statistical analyses were performed by applying SPSS software (version 23.0, SPSS Inc., Chicago, IL, USA), GraphPad Prism software (version, 8.0La Jolla, CA, USA) and MedCalc software (version 15.2.2). Data was exhibited as mean ± standard deviation (SD). Student’s t-test was used to investigate continuous variables. Multiple hypothesis test was assessed by using Monte Carlo method. The Person χ2 test was applied to examine correlations. The survival curves were plotted using Kaplan-Meier method and tested by log-rank test. Subsequently, Cox proportional hazards regression model (enter method) was utilized to evaluation of potential independent prognostic factors. Potential confounders with *P* values less than 0.05 in univariate regression analyses were selected for multivariate regression models. A two-tailed P value < 0.05 was considered statistically significant.

## Results

### NCSTN is frequently upregulated in HCC tissues and correlates with the patient prognosis

**Fig. 1:**
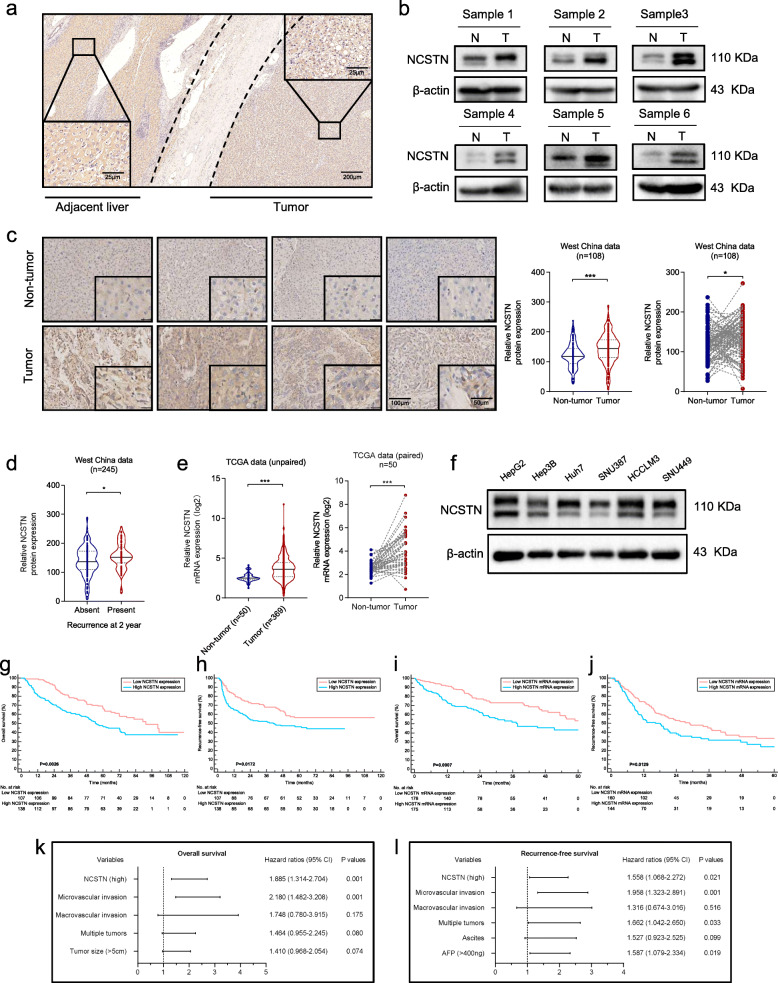
NCSTN is overexpressed in HCC and serves as a prognostic predictor. **a** Representative immunohistochemistry images of NCSTN expression in HCC and adjacent liver tissues. Scale bars: 25 μm (lower left and upper right corner), 200 μm (lower right corner). **b** Representative images of NCSTN expression obtained by western blotting analysis in HCC tumor tissues compared to matched non-tumor tissues. T, HCC tumor tissue; N, paired non-tumor tissue. Loading control was assessed by β-actin. **c** Representative images of NCSTN expression with different levels in HCC tumor tissues and paired non-tumor tissues. Scale bars, 100 μm (left), 25 μm (right). **d** Relative NCSTN protein expression in HCC patients with or without early recurrence. **e** Relative NCSTN mRNA expression in tumor tissues and non-tumor tissues. **f** Relative protein expression of NCSTN in HCC tumor cell lines obtained by western blotting analysis. **g, h** Kaplan-Meier survival curves showed correlation between NCSTN expression and overall survival or recurrence-free survival in HCC patients from West China Hospital dataset. **i, j** Kaplan-Meier survival curves showed correlation between NCSTN expression and overall survival or recurrence-free survival in HCC patients from TCGA dataset. **k, l** Multivariate analyses showed prognostic factors for overall survival and recurrence-free survival. HCC, hepatocellular carcinoma; TCGA, The Cancer Genome Atlas. *p < 0.05, **p < 0.01, ***p < 0.001

To investigate the potential role of NCSTN in HCC, we first evaluated the expression level of NCSTN in 108 matched HCC and adjacent samples. Immunohistochemistry (IHC) staining and western blot analysis revealed an enhanced expression level of NCSTN in HCC tissues compared to that in adjacent normal tissues (Fig. 1a-c). IHC analysis also demonstrated an elevated expression of NCSTN in recurrence cases compared with non-recurrent ones (Fig. 1d). Subsequently, the expression of NCSTN was analyzed using The Cancer Genome Atlas (TCGA) dataset. Consistently, NCSTN expression was much higher in tumor than in adjacent liver tissues at the mRNA level (Fig. 1e). In addition, the expression levels of endogenous NCSTN were detected in six different types of HCC cell lines, HepG2, Hep3B, Huh7, SNU387, HCCLM3 and SNU449. The results demonstrated that the NCSTN expression levels were lower in Hep3B and Huh7 than that in HCCLM3 and SNU449 (Fig. 1f).

To further determine whether the NCSTN expression level in HCC tissues was associated with patient prognosis, we measured the expression level of NCSTN in a cohort of 245 HCC tissues (including the previous 108 samples) using tissue microarray. Patients were stratified into high NCSTN expression group (*n* = 138) and low NCSTN expression group (*n* = 107) according to expression of NCSTN in HCC tissues. Table 1 showed the baseline characteristics of included 245 patients. A higher NCSTN expression level was significantly associated with higher serum AFP level, tumor size as well as poorer tumor differentiation, indicating that the NCSTN was possibly correlated to HCC growth and progression. Kaplan-Meier survival curves revealed that HCC patients in high NCSTN expression group were significantly associated with worse overall survival (OS, *p* = 0.0028) and recurrence-free survival (RFS, *p* = 0.0172) compared to those with lower NCSTN expression (Fig. 1g-h). Kaplan-Meier analysis of patients from TCGA dataset confirmed our results (Fig. 1i-j). In univariate analysis, serum AFP level, status of ascites, tumor size, tumor number, macrovascular invasion, microvascular invasion and NCSTN expression were identified as potential variables correlated to OS or RFS of HCC patients (Table 2). They were subsequently analyzed in multivariate regression. The results illustrated that high NCSTN expression and microvascular invasion were independent risk factors for OS, whereas high NCSTN expression, microvascular invasion, multiple tumors and elevated serum AFP level were independent risk factors for RFS (Fig. 1k-l, Table 3).

**Table 1:** Baseline characteristics of included patients

Variables	All patients (*n* = 245)	Low NCSTN (*n* = 107)	High NCSTN (*n* = 138)	*P* value
Patient factors/Laboratory parameters				
Age [year, mean (SD)]	51.6 (12.4)	51.5 (12.4)	51.8 (12.4)	0.825
Male gender, n (%)	201 (82.0)	83 (77.6)	118 (85.5)	0.132
HBsAg, [positive, n (%)]	210 (85.7)	95 (88.8)	115 (83.3)	0.228
AFP ≥ 400, n (%)	93 (38.0)	32 (29.9)	61 (44.2)	0.022
Ascites, n (%)	30 (12.2)	12 (11.2)	18 (13.0)	0.665
Histological and gross features of tumors				
Tumor size, [cm, mean (SD)]	5.7 (3.3)	4.3 (3.2)	6.3 (3.3)	< 0.001
Solitary tumor, n (%)	204 (83.3)	92 (86.0)	112 (81.1)	0.332
Tumor differentiation				0.027
Well/Moderate	143 (58.4)	69 (64.5)	74 (53.6)	
Poor	102 (41.6)	38 (35.5)	64 (49.5)	
Macrovascular invasion, n (%)	9 (3.7)	4 (3.7)	5 (3.6)	0.962
Microvascular invasion, n (%)	80 (32.7)	32 (29.9)	48 (34.8)	0.420
Cirrhosis, n (%)	153 (62.4)	66 (61.7)	87 (63.0)	0.827
TNM stage, n (%)				0.060
I- II	186 (75.9)	86 (80.4)	100 (72.5)	
III	59 (24.1)	21 (19.6)	38 (27.5)	
BCLC stage				0.493
A	198 (80.8)	83 (77.6)	115 (83.3))	
B	36 (14.7)	19 (17.8)	18 (13.0)	
C	10 (4.1)	5 (4.7)	5 (3.6)	
Prognostic outcome				
Overall survival, months, mean (95% CI)	31.5 (29.5, 33.6)	36.0 (32.7, 39.3)	28.1 (25.6, 30.6)	

*TNM* Tumor-node-metastasis, *SD* Standard deviation, *CI* Confidence interval

**Table 2:** Prognostic factors for overall survival and recurrence-free survival by the univariate Cox proportional hazards regression model

Variables	Overall survival			Recurrence-free survival		
	HR	95% CI	***P***	HR	95% CI	***P***
Age	0.998	0.979–1.018	0.872	0.998	0.984–1.013	0.807
Gender (F/M)	0.922	0.481–1.769	0.807	0.920	0.574–1.475	0.729
HBsAg	1.581	0.908–2.753	0.105	1.394	0.798–2.437	0.243
AFP (≥400/< 400)	1.335	0.943–1.891	0.104	1.799	1.252–2.586	0.001
Ascites (+/−)	1.057	0.451–1.986	0.884	1.693	1.025–2.797	0.040
Tumor size (> 5/≤5)	1.755	1.246–2.530	0.002	1.440	0.998–2.077	0.051
Multiple tumors	1.754	1.161–2.652	0.008	1.734	1.114–2.698	0.015
Differentiation (Poor/Well-Moderate)	1.188	0.842–1.678	0.327	1.081	0.751–1.560	0.671
TNM stage (III/I-II)	1.325	0.902–1.946	0.151	1.456	0.973–2.177	0.067
Macrovascular invasion	3.039	1.408–6.556	0.005	2.739	1.268–5.915	0.010
Microvascular invasion	2.621	1.846–3.721	0.001	2.337	1.621–3.369	0.001
Cirrhosis	1.191	0.832–1.707	0.339	1.174	0.803–1.715	0.407
NCSTN (high/low)	1.721	1.203–2.460	0.003	1.566	1.080–2.272	0.017

*TNM* Tumor-node-metastasis, *HR* Hazard ratio, *CI* Confidence interval, *F* Female, *M* Male

**Table 3:** Independent prognostic factors for overall survival and recurrence-free survival by the multivariate Cox proportional hazards regression model

Variables	Overall survival			Recurrence-free survival		
	HR	95% CI	***P***	HR	95% CI	***P***
AFP (≥400/< 400)				1.587	1.079–2.334	0.019
Ascites (+/−)				1.527	0.923–2.525	0.099
Tumor size (> 5/≤5)	1.410	0.968–2.054	0.074			
Multiple tumors	1.464	0.955–2.245	0.080	1.662	1.042–2.650	0.033
Macrovascular invasion	1.748	0.780–3.915	0.175	1.316	0.674–3.016	0.516
Microvascular invasion	2.180	1.482–3.208	0.001	1.958	1.323–2.891	0.001
NCSTN (high/low)	1.885	1.314–2.704	0.001	1.558	1.068–2.272	0.021

*HR* Hazard ratio, *CI* Confidence interval

Collectively, these finding suggested that NCSTN is significantly upregulated in HCC and may be involved in HCC progression. Moreover, NCSTN is a potential prognostic predictor for HCC patients.

### NCSTN promotes HCC cell growth and metastasis in vitro and vivo

**Fig. 2:**
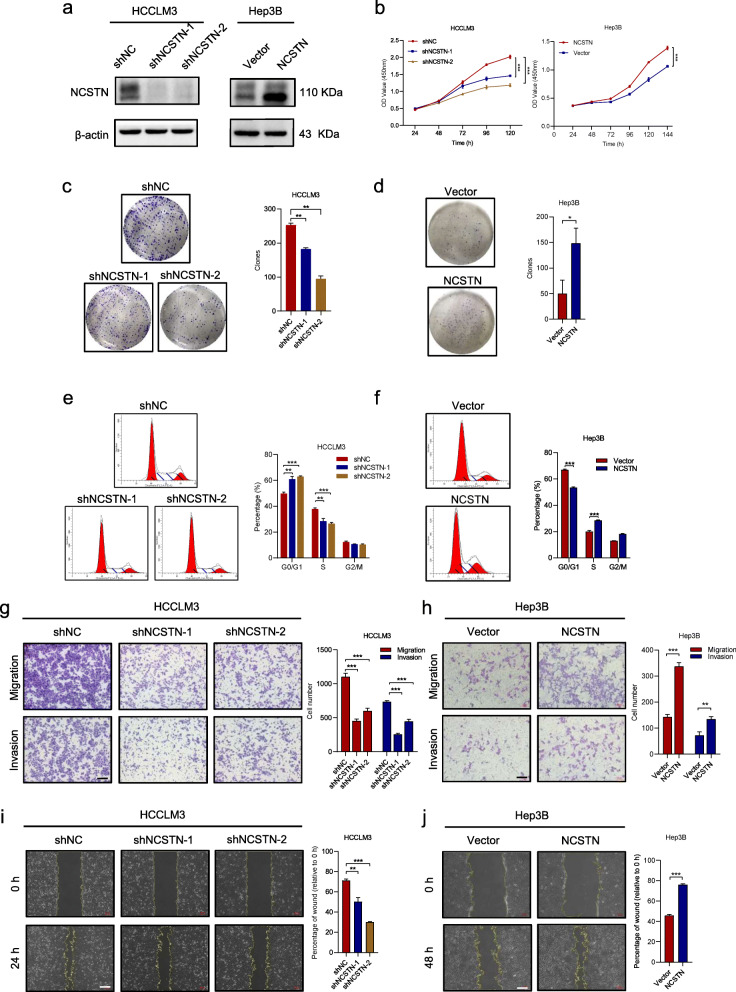
NCSTN promotes HCC cell growth and metastasis in vitro. **a** The effects of NCSTN knockdown and overexpression were examined by western blotting analysis in HCCLM3 and Hep3B cells. Loading control was assessed by β-actin. **b** CCK8 assays showed NCSTN depletion inhibited cell growth of HCCLM3 and NCSTN overexpression promoted cell growth of Hep3B. **c, d** Colony formation assays showed colony numbers in HCC cells with NCSTN depletion or overexpression. **e, f** The cell cycle assays showed that NCSTN depletion increased the G0/G1 fraction and decreased the S and G2/M fraction in HCCLM3 cells, whereas NCSTN overexpression decreased the G0/G1 fraction and increased the S and G2/M fraction in Hep3B cells. **g, h** The migration and invasion capacity was determined in the indicated HCC cells. Scale bar, 100 μm. **i, j** Wound healing assays showed the migration capacity of indicated HCC cells. Scale bar, 100 μm. HCC, hepatocellular carcinoma; CCK8, cell counting kit-8. *p < 0.05, **p < 0.01, ***p < 0.001

To further elucidate the functions of NCSTN in HCC cell growth and metastasis, we knocked down NCSTN in HCCLM3 and SNU449 cells, which exhibited relatively high endogenous NCSTN levels. Additionally, we established stable overexpression cell lines via a lentiviral infection in Hep3B and Huh7 cells, which exhibited relatively low endogenous NCSTN levels (Fig. 2a and Additional file 4: Fig. S1a). NCSTN silencing significantly inhibited cell proliferation and cell cycle in HCCLM3 and SNU449, while NCSTN overexpression promoted cell growth (Fig. 2b and Additional file 4: Fig. S1b). In addition, the colony-forming ability was markedly weakened in NCSTN silencing cells (Fig. 2c and Additional file 4: Fig. S1c), whereas significantly up-regulated by overexpression of NCSTN in Hep3B and Huh7 cells (Fig. 2d and Additional file 4: Fig. S1d). The cell cycle assay revealed that NCSTN depletion markedly increased the G0/G1 fraction and decreased the S and G2/M fraction in HCCLM3 and SNU449 cells, while NCSTN overexpression decreased the G0/G1 fraction and increased the S and G2/M fraction in Hep3B and Huh7 cells (Fig. 2e-f and Additional file 4: Fig. S1e-f). The transwell migration, invasion and wound-healing migration assays revealed a marked suppression in motility of HCC cells after knockdown of NCSTN. Consistently, overexpression of NCSTN promoted cell migration and invasion capability (Fig. 2g-j and Additional file 4: Fig. S1g-j).

**Fig. 3:**
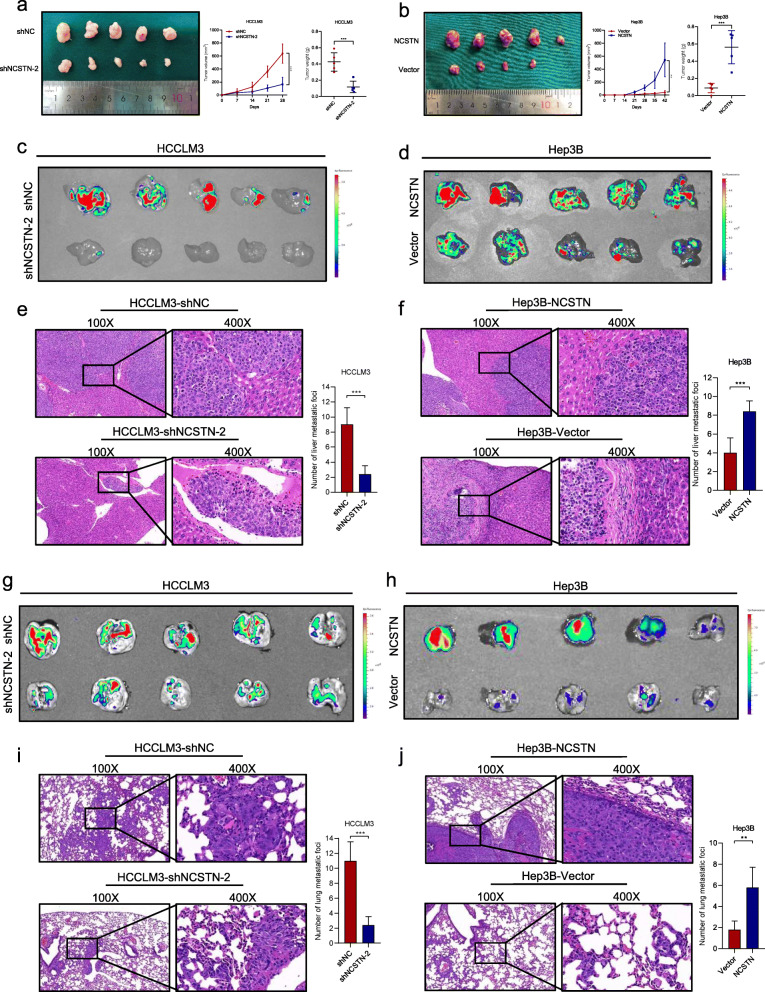
NCSTN promotes tumor growth and metastasis in vivo. **a, b** Tumor volume and weight of subcutaneous xenografts in nude mice injected with indicated HCCLM3 and Hep3B cells. **c, d** Fluorescence images of intrahepatic metastatic foci in liver orthotopic-implanted HCC models with indicated HCC cells. **e, f** Representative images of corresponding hematoxylin and eosin staining of liver orthotopic-implanted HCC models. Scale bars, 100 μm. **g, h** Fluorescence images of lung metastatic foci in lung metastasis models with indicated HCCLM3 and Hep3B cells. **i, j** Representative images of corresponding hematoxylin and eosin staining of lung metastasis models. Scale bars, 100 μm. *p < 0.05, **p < 0.01, ***p < 0.001

We then examined the effect of NCSTN on tumorigenicity in vivo. HCCLM3 cells infected with shNCSTN-2 or shNC, and Hep3B cells infected with NCSTN or empty vector were subcutaneously implanted into nude mice. The tumor volume and weight were dramatically higher in HCCLM3-shNC and Hep3B-NCSTN compared to those of corresponding HCCLM3-shNCSTN-2 and Hep3B-Vector (Fig. 3a-b), indicating that NCSTN promoted tumor growth in vivo. Next, to further evaluate in vivo functions of NCSTN in HCC cell metastasis, liver orthotopic-implanted HCC models and lung metastasis models were established. Compared to HCCLM3-shNC group, NCSTN silencing group was associated with significantly decreased fluorescence signal intensities, whereas Hep3B-NCSTN group was associated with higher fluorescence signal intensities than those of Hep3B-Vector group (Fig. 3c-d). The results of hematoxylin and eosin (HE) staining and IHC staining of Ki-67 showed decreased size and number of intrahepatic metastatic foci in HCCLM3-shNCSTN-2 and Hep3B-Vector group, indicating that NCSTN remarkably promoted intrahepatic metastasis ability of HCC cells (Fig. 3e-f and Additional file 5: Fig. S2a-b). Similarly, in the lung metastasis models, the fluorescence signal intensities and number of metastatic foci were dramatically inhibited in HCCLM3-shNCSTN-2 and Hep3B-Vector group compared to those of HCCLM3-shNC and Hep3B-NCSTN group (Fig. 3g-j and Additional file 5: Fig. S2c-d). Collectively, these results suggested NCSTN facilitated HCC cell growth and metastasis in vitro and in vivo.

### NCSTN induces EMT in HCC cells

**Fig. 4:**
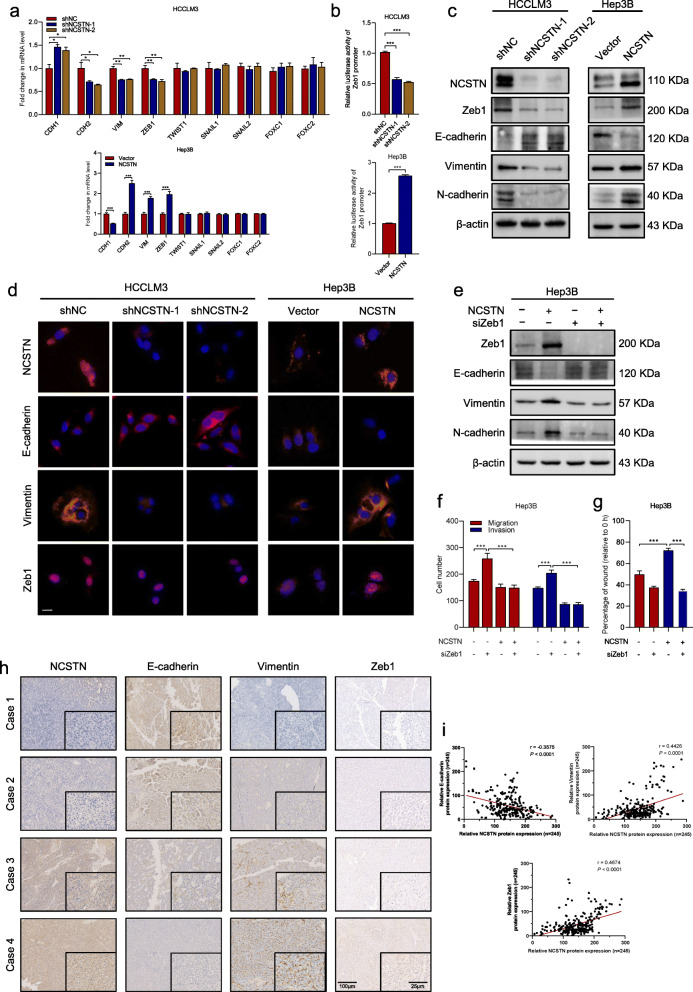
NCSTN induces EMT in HCC cells via activation of Zeb1. **a** The mRNA expression levels of EMT markers CDH1, CDH2, VIM, ZEB1, SNAIL1, SNAIL2, FOXC1, FOXC2 and TWIST1 in the indicated HCC cells. **b** Luciferase reporter assays showed relative luciferase activity of Zeb1 promoter in indicated cells. **c** The expression levels of four EMT-related proteins E-cadherin, Zeb1, Vimentin and N-cadherin in the indicated cells. Loading control was assessed by β-actin. **d** Immunofluorescence assays showed the expression of NCSTN, E-cadherin, Vimentin and Zeb1 in the indicated cells. Scale bars, 100 μm. **e** The expression levels of EMT-related markers E-cadherin, Vimentin and N-cadherin in the indicated cells co-transfected with siZeb1 or negative control. Loading control was assessed by β-actin. **f** The migration and invasion capacity was examined in the indicated HCC cells co-transfected with siZeb1 or negative control. g Wound healing assays showed the migration capacity of indicated HCC cells. **h** Representative images of EMT-related markers E-cadherin, Vimentin and N-cadherin in HCC tissues by immunohistochemical staining. Scale bar, 100 μm. **i** Correlation analyses of protein expression levels between NCSTN and E-cadherin, Vimentin or nuclear Zeb1 in HCC tissues. *p < 0.05, **p < 0.01, ***p < 0.001

To elucidate the potential molecular mechanisms underlying NCSTN in promoting HCC metastasis, we first investigated whether EMT markers were regulated. Analysis of mRNA expression level of the epithelial marker E-cadherin (CDH1) and mesenchymal markers N-cadherin (CDH2), vimentin (VIM), ZEB1, SNAIL1, SNAIL2, FOXC1, FOXC2 and TWIST1 indicated that overexpression NCSTN induced EMT. Consistently, knockdown of NCSTN increased CDH1 and reduced CDH2, VIM and ZEB1 (Fig. 4a). However, no significant change was observed in EMT transcription factors TWIST1, SNAIL1, SNAIL2, FOXC1 and FOXC2. To determine the transcriptional activation of NCSTN on Zeb1 gene promoter, cells were transfected with Zeb1 promoter luciferase reporter. NCSTN silencing markedly reduced the Zeb1 promoter activity, whereas enhanced NCSTN increased its activity (Fig. 4b). In addition, the western blot assay confirmed NCSTN overexpression significantly inhibited the protein expression of E-cadherin and increased Vimentin, N-cadherin and Zeb1 (Fig. 4c). The immunofluorescence assay confirmed that NCSTN depletion significantly upregulated expression of E-cadherin, and diminished expression of Vimentin and Zeb1 (Fig. 4d). To further whether NCSTN induced EMT via upregulation of ZEB1, we silenced Zeb1 in Hep3B cells. The EMT-related proteins were rescued in NCSTN-overexpressed Hep3B cells (Fig. 4e). Furthermore, the NCSTN-mediated cell motility and invasion was abolished in Zeb1-depletion Hep3B cells (Fig. 4f-g).

Further, we examined expression of EMT markers in HCC tissues via IHC analysis. In cases with higher expression of NCSTN, reduced E-cadherin and increased Vimentin, Zeb1 expression were observed. Subsequent correlation analysis revealed significantly negative correlation of NCSTN with E-cadherin expression (*r* = − 0.3575, *p* < 0.001), positive correlation of NCSTN with Vimentin expression (*r* = 0.4426, *p* < 0.001) and Zeb1 expression (*r* = 0.4674, *p* < 0.001, Fig. 4h-i and Additional file 5: Fig. S2e). The correlation between NCSTN and EMT markers were confirmed using TCGA LIHC dataset (Additional file 5: Fig. S2f). Altogether, these results suggested NCSTN induced EMT in HCC via upregulation of Zeb1.

### NCSTN promotes EMT process via nuclear translocation of β-catenin

**Fig. 5:**
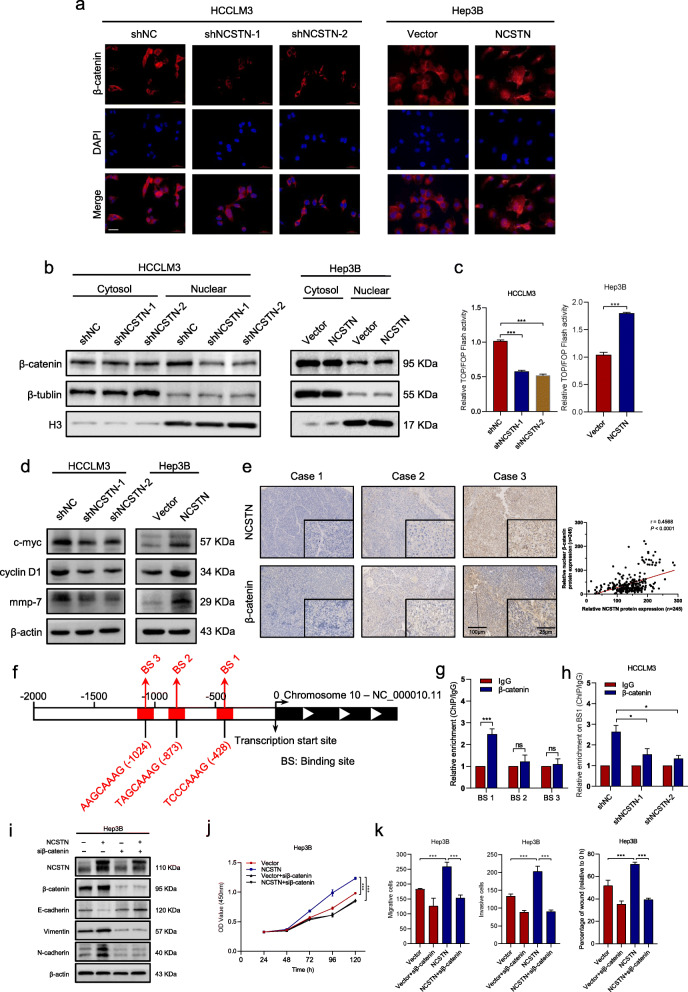
NCSTN promotes nuclear translocation of β-catenin. **a** Immunofluorescence assays showed NCSTN increased nuclear translocation of β-catenin. Scale bars, 100 μm. **b** Subcellular fractionation assays showed the expression of β-catenin in the cytosol and nuclear of indicated cells. Loading control was assessed by β-tublin and Histone H3. **c** TOP/FOP Flash assays showed the transcriptional activity of β-catenin in the indicated HCCLM3 and Hep3B cells. **d** The expression levels of β-catenin target protein c-myc, cyclin D1 and mmp-7 in the indicated cells. **e** Representative immunohistochemical staining images of NCSTN and β-catenin in HCC tissues (left), correlation between NCSTN expression and nuclear β-catenin expression (right). Scale bars, 100 μm (left), 25 μm (right). **f** Schematic outlines of the predicted binding sites of β-catenin on Zeb1 gene promoter region. **g** ChIP assays of the enrichment of β-catenin on BSs in the promoter region of Zeb1 relative to IgG. **h** ChIP assays showed relative enrichment of β-catenin on BS1 in the Zeb1promoter region in the indicated cells. **i** The expression levels of EMT-related protein in the indicated Hep3B cells co-transfected with or without siβ-catenin. **j** CCK8 assays showed that NCSTN-mediated cell growth could be rescued in Hep3B cells co-transfected with siβ-catenin. **k** The migration and invasion capacity was rescued in the indicated Hep3B cells co-transfected with siβ-catenin. BS, binding sites. *p < 0.05, **p < 0.01, ***p < 0.001

β-catenin, the core element of the Wnt/β-catenin signaling pathway, has been reported frequently activated in HCC metastasis and was closely related to EMT process [14–16]. Therefore, we further explored whether β-catenin signaling pathway was involved in NCSTN-regulated EMT. First, we examined the correlation between NCSTN and CTNNB1 using TCGA dataset. The result revealed that mRNA expression of NCSTN was significantly correlated to CTNNB1 (*r* = 0.64, *p* < 0.001, Additional file 6: Fig. S3a). Then immunofluorescence assays were performed to detect subcellular localization changes of β-catenin. NCSTN silencing significantly redistributed nuclear β-catenin into cytoplasm in HCCLM3-shNCSTN cells. In contrast, overexpression of NCSTN increased nuclear translocation of β-catenin in Hep3B-NCSTN cells (Fig. 5a). Further subcellular fractionation assays showed that the expression of nuclear β-catenin was significantly enhanced in NCSTN overexpression cells and decreased in NCSTN silencing cells, without alteration of cytosolic β-catenin levels (Fig. 5b). Then the luciferase reporter assays were used to explore the transcriptional activity of β-catenin signaling, which revealed that silencing of NCSTN suppressed the transcriptional activity in HCCLM3 cells whereas, while NCSTN overexpression increased the activity in Hep3B cells (Fig. 5c). Consistent with results of TOP/FOP Flash assays, NCSTN depletion significantly reduced the expression levels of β-catenin target genes GS, TBX3, AXIN2 and Survivin in HCCLM3 cells, while overexpression of NCSTN showed the opposite results in Hep3B cells (Additional file 6: Fig. S3b). The correlation between NCSTN and β-catenin, GS, AXIN2 and TBX3, were confirmed using TCGA LIHC dataset (Additional file 6: Fig. S3c). The results were confirmed by western blot assays (Fig. 5d), suggesting that NCSTN might promote cell growth through upregulating the oncogenic targets of β-catenin, such as GS, TBX3, AXIN2 and Survivin. Next, we evaluated the correlation between expression level of NCSTN and nuclear β-catenin in HCC tissues. IHC analysis showed higher nuclear β-catenin expression in cases with elevated NCSTN expression (*r* = 0.4568, *p* < 0.001, Fig. 5e). We also examined the expression level of nuclear β-catenin in liver and lung metastastic foci, nuclear β-catenin expression was markedly enhanced in Hep3B-NCSTN group and downregulated in HCCLM3-shNCSTN-2 group compared to those of control groups (Additional file 6: Fig. S3d-g).

To determine whether β-catenin showed regulation on Zeb1 promoter activity, bioinformatics analyses were performed to predict the potential biding site (BS) of β-catenin on Zeb1 promoter region (Fig. 5f). Subsequent ChIP assays confirmed significantly high enrichment of β-catenin on BS1 in the promoter region of Zeb1 (Fig. 5g), which could be significantly reduced by depletion of NCSTN (Fig. 5h).

To further determine whether NCSTN induced EMT via β-catenin signaling, we knocked down the expression of β-catenin in Hep3B-NCSTN and Hep3B-Vector cells. β-catenin silencing could significantly reduce the expression of β-catenin target genes (Additional file 7: Fig. S4a-b). Knockdown of β-catenin could eliminate NCSTN-induced EMT changes (Fig. 5i). Additionally, the NCSTN-upregulated cell proliferation, migration and invasion could also be abrogated after knockdown of β-catenin (Fig. 5j-k and Additional file 7: Fig. S4c-d). Taken together, these results suggested NCSTN promoted nuclear translocation of β-catenin, thus inducing cell growth and EMT in HCC cells.

### NCSTN regulates β-catenin through notch/AKT/GSK-3β signaling pathway in HCC cells

**Fig. 6:**
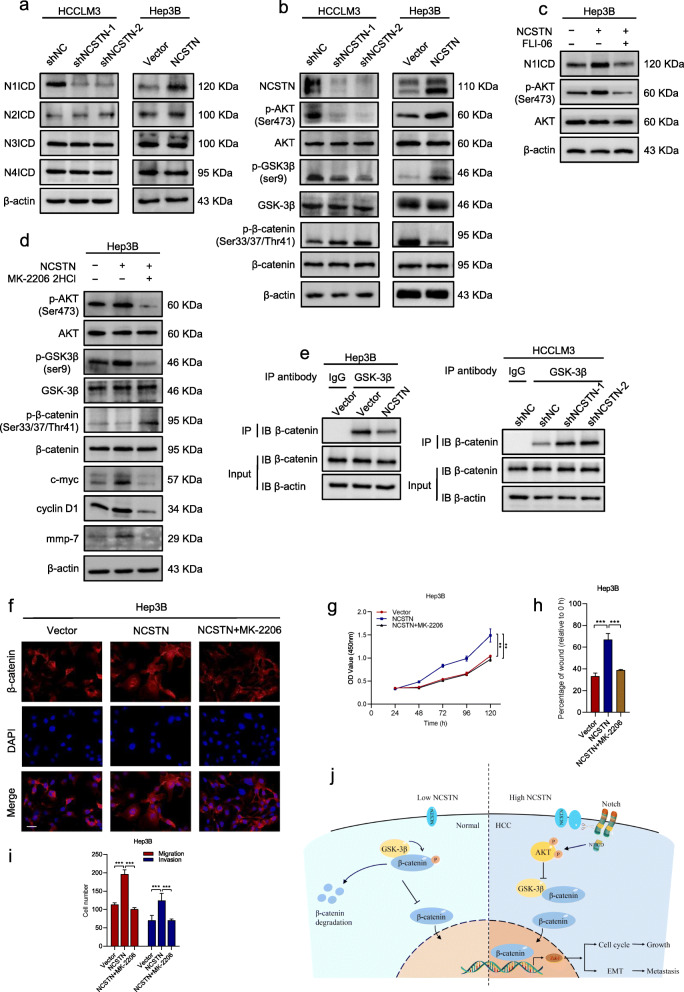
NCSTN regulates β-catenin through Notch/AKT/GSK-3β signaling pathway. **a** The expression of N1ICD, N2ICD, N3ICD, and N4ICD in the indicated HCCLM3 and Hep3B cells. **b** Representative immunoblot analyses of total AKT, phosphorylated AKT (Ser473), GSK-3β, phosphorylated GSK-3β (ser9), β-catenin and phosphorylated β-catenin (Ser33/37/Thr41) in the indicated HCC cells. **c** The expression of N1ICD, AKT and p-AKT in the indicated Hep3B cells, treated with FLI-06 (the Notch inhibitor, 5 μM, 24 h). **d** Representative immunoblot analyses of AKT, p-AKT (Ser473), GSK-3β, p-GSK-3β (ser9), β-catenin and p-β-catenin (Ser33/37/Thr41) in the indicated Hep3B cells, treated with MK-2206 2HCl (the AKT inhibitor, 10 μM, 24 h). **e** Co-IP assays showed NCSTN regulated the interaction between GSK-3β and β-catenin in the indicated cells. **f** Immunofluorescence assays showed NCSTN-mediated nuclear translocation of β-catenin was rescued in cells treated with MK-2206 2HCl. **g** CCK8 assays showed that NCSTN-mediated cell growth could be rescued in Hep3B cells treated with MK-2206 2HCl. **h, i** The migration and invasion capacity was rescued in cells treated with MK-2206 2HCl. **j** The propose model showing the regulatory landscape of NCSTN/Notch/AKT/β-catenin signaling axis in promoting cell growth and metastasis of HCC. Loading control was evaluated by β-actin. *p < 0.05, **p < 0.01, ***p < 0.001

Previous study has revealed that NCSTN regulated breast cancer stem cell properties and growth via Notch/AKT pathway [8]. To determine whether NCSTN-induced phenotypes and nuclear translocation of β-catenin in HCC cells was also dependent on Notch/AKT signaling pathway, we first evaluated the expression of Notch intracellular domain (NICD) and phosphorylated AKT (p-AKT) in HCCLM3 and Hep3B cells. Strikingly, depletion of NCSTN significantly repressed the expression of Notch target genes HES1, MAML1, MYC and P21, while NCSTN overexpression promoted these genes (Additional file 8: Fig. S5a). Further WB assays confirmed that activated Notch1 was reduced in HCCLM3-shNCSTN cells and increased in Hep3B-NCSTN cells compared with those of control cells, whereas N2ICD, N3ICD and N4ICD remained unchanged (Fig. 6a). Furthermore, the expression of p-AKT decreased in NCSTN-silencing cells and elevated in overexpressing cells, while total AKT remained unchanged (Fig. 6b). We then repressed Notch signaling pathway by using FLI-06 in Hep3B-NCSTN cells. The NCSTN-induced upregulated p-AKT was abrogated by inhibition of Notch pathway (Fig. 6c), suggesting NCSTN might regulate AKT signaling through cleaving Notch1.

Activated GSK-3β-mediated phosphorylation and subsequent degradation of β-catenin is an important manner of β-catenin regulation. In addition, the GSK-3β is negatively regulated by active AKT signaling pathway. To investigate whether NCSTN-mediated β-catenin activation is regulated by AKT/GSK-3β manner, the expression of GSK-3β and phosphorylated β-catenin (p-β-catenin) was evaluated. The expression of inactivate GSK-3β (p-GSK-3β) was markedly reduced, while p-β-catenin was accumulated in HCCLM3-shNCSTN cells. In contrast, opposite results were observed in NCSTN overexpressed HCC cells (Fig. 6b). Besides, we performed co-IP assays to examine the formation of GSK-3β/β-catenin complex in HCCLM3 and Hep3B cells. The results shown that the amount of GSK-3β/β-catenin complex was dramatically increased in NCSTN depletion cells, whereas it was reduced in NCSTN overexpression cells (Fig. 6e and Additional file 8: Fig. S5b). We then used AKT specific inhibitor MK-2206 2HCl to repress AKT signaling. In the presence of MK-2206, the expression level of NCSTN-mediated p-GSK-3β was eliminated, whereas p-β-catenin was accumulated in Hep3B cells (Fig. 6d). The immunofluorescence assays showed a significantly enhanced expression of nuclear β-catenin in NCSTN overexpression Hep3B cells. However, inhibition of AKT signaling markedly redistributed nuclear β-catenin into cytoplasm (Fig. 6f). NCSTN-mediated promotion of cell proliferation, migration and invasion were eliminated by inhibition of AKT signaling (Fig. 6g-i and Additional file 8: Fig. S5c-d). The correlation between NCSTN and nuclear NOTCH1 as well as p-AKT (Ser 473) were confirmed using human HCC collections (Additional file 8: Fig. S6a-b) and subcutaneous xenografts (Additional file 9: Fig. S6c). Collectively, the above findings suggested NCSTN regulated β-catenin activation via stimulating Notch/AKT/ GSK-3β signaling pathway.

## Discussion

Accumulating studies have revealed that γ-Secretase played a vital role in tumorigenesis and metastasis of multiple malignancies [17–19]. Recent clinical trials showed promising clinical benefits of γ-Secretase inhibitors in patients with advanced tumors [20–22]. However, the γ-Secretase inhibitors inhibited the catalytic activity of Presenilin, which was responsible for several non-selective inhibition of the wide range of γ-Secretase substrates. Several phase I and II clinical trials have reported accompanying dose-limiting toxicity profiles of γ-Secretase inhibitors, primarily concerning gastrointestinal goblet cells hyperplasia [23, 24]. Structurally, NCSTN is the only γ-Secretase component that contains a large extracellular domain, which holds the potential to serve as a drug target for monoclonal antibody (mAb) therapy. Filipovic et al. have developed mAbs against extracellular domain of NCSTN in invasive breast cancer cells, and demonstrated targeting NCSTN as a promising mode of treatment for those with few therapeutic options [10]. Here we demonstrate that NCSTN is also a candidate therapeutic target for HCC.

In this study, we highlight the critical role of NCSTN, a core subunit of γ-Secretase, in HCC progression. Mounting evidence have revealed that EMT was of critical importance in initiation of metastasis and invasion, a binary process involving the conversion of tumor cells from epithelial to mesenchymal status, as well as acquisition of invasive capacity [25, 26]. A series of transcriptional factors could drive EMT process and thereby tumor-initiation, metastasis, response to chemotherapy and immune evasion [27, 28]. Our previous work has demonstrated Zeb1 as a vital activator in thioredoxin domain-containing protein 12-mediated EMT process of HCC [29]. In the present work, we demonstrated that EMT process was dispensable for NCSTN-regulated HCC cell growth and metastasis, and Zeb1 was the key activator involved. In addition, our work also focused on the underlying molecular mechanisms of NCSTN-regulated EMT and aggressive features.

It has been widely reported that β-catenin signaling pathway played a crucial role in EMT process via nuclear translocation of β-catenin [30, 31]. The dysregulation of β-catenin was involved in HCC progression. Additionally, CTNNB1 mutation was frequently detected in HCC tissues compared to those of adjacent liver [32]. The data in this study identified NCSTN as the upstream regulator of β-catenin and promoted its nuclear translocation, thereby induced Zeb1-mediated EMT process, resulting in malignant phenotype. The activated β-catenin translocated into nuclear and formed a transcriptional activation complex with TCF4, then bound to Zeb1 promoter to activate its transcription [33].

This work revealed that NCSTN overexpression activated Notch1, whereas its depletion achieved an opposite effect. The intracellular domain of Notch1 (N1ICD) was released from the membrane tether, and then induced AKT phosphorylation. These observations were consistent with the conclusions of previous studies that Notch signaling could induce an autocrine loop to activate AKT [34]. The activated AKT promoted the phosphorylation of GSK-3β on Ser9 and thereby inactivated its activity. The inactivation of GSK-3β inhibited formation of GSK-3β/β-catenin complex and β-catenin degradation, resulting in nuclear translocation of β-catenin.

## Conclusions

In summary, this study illustrated that reinforced NCSTN expression promoted EMT in HCC via upregulation of Zeb1. NCSTN increased cleavage of Notch1 and AKT phosphorylation as well as decreased GSK-3β/β-catenin complex. The inactivation of GSK-3β inhibited the β-catenin degradation and promoted nuclear translocation of β-catenin to initiate the transcription of Zeb1, resulting in HCC cell growth and metastasis (Fig. 6j). Thus, our work identified the vital role of NCSTN in HCC progression and the underlying molecular mechanisms, and importantly, laid the basis for future NCSTN-targeting therapeutic strategies in HCC.

## Supplementary information

**Additional file 1: Table S1.** Primary antibodies used in this study. **Table S2.** Primers used in this study. **Table S3**. Sequences of siRNA and shRNA used in this study.

**Additional file 2: Supplementary figure legends**

**Additional file 3.** The raw data of gene expression in TCGA LIHC dataset.

**Additional file 4: Figure S1.** NCSTN promotes HCC cell growth and metastasis in vitro.

**Additional file 5: Figure S2.** Immunohistochemical staining of metastatic foci and EMT markers.

**Additional file 6: Figure S3.** NCSTN promoted activation of β-catenin.

**Additional file 7: Figure S4.** Biological effect of NCSTN is rescued by knockdown of β-catenin in indicated cells.

**Additional file 8: Figure S5.** NCSTN regulates β-catenin through Notch/AKT/GSK-3β signaling pathway.

**Additional file 9: Figure S6.** Correlation between NCSTN and p-AKT, nuclear NOTCH1 as well as EMT markers in human HCC collections and subcutaneous xenografts.

## Abbreviations

NCSTN: Nicastrin
HCC: Hepatocellular carcinoma
EMT: Epithelial-mesenchymal transition
IHC: Immunohistochemistry
WB: Western blotting
TCGA: The Cancer Genome Atlas
OS: Overall survival
siRNA: Small interfering RNA
CCK-8: Cell counting kit-8
IF: Immunofluorescence
ChIP: Chromatin immunoprecipitation
Co-IP: Co-immunoprecipitation
HE: Hematoxylin and eosin
NICD: Notch intracellular domain
p-AKT: Phosphorylated AKT
mAb: Monoclonal antibody
