# Correction: NCSTN promotes hepatocellular carcinoma cell growth and metastasis via β-catenin activation in a Notch1/AKT dependent manner

**DOI:** 10.1186/s13046-023-02617-0

**Published:** 2023-02-15

**Authors:** Hui Li, Tian Lan, Lin Xu, Hailing Liu, Jinju Wang, Jiaxin Li, Xiangzheng Chen, Jiwei Huang, Xuefeng Li, Kefei Yuan, Yong Zeng, Hong Wu

**Affiliations:** 1 Department of Liver Surgery, Liver Transplantation Division, Laboratory of Liver Surgery, West China Hospital, Sichuan University, Chengdu, 610041 China; 2 Laboratory of Liver Surgery, West China Hospital, Sichuan University, Chengdu, 610041 China; 3 School of Basic Medical Sciences, Guangzhou Medical University, Guangzhou, 511436 China; 4 Shenzhen Luohu People’s Hospital, The Third Affiliated Hospital of Shenzhen University, Shenzhen, 518001 China

**Correction:** ***J Exp Clin Cancer Res*** **39, 128 (2020)**

**https://doi.org/10.1186/s13046-020-01638-3**

Following publication of the original article [1], author identified an error in Fig. 2, specifically:

- Figure 2h - the cell migration assay of Hep3B-Vector group

The correct figure is presented below:

**Fig. 2:**
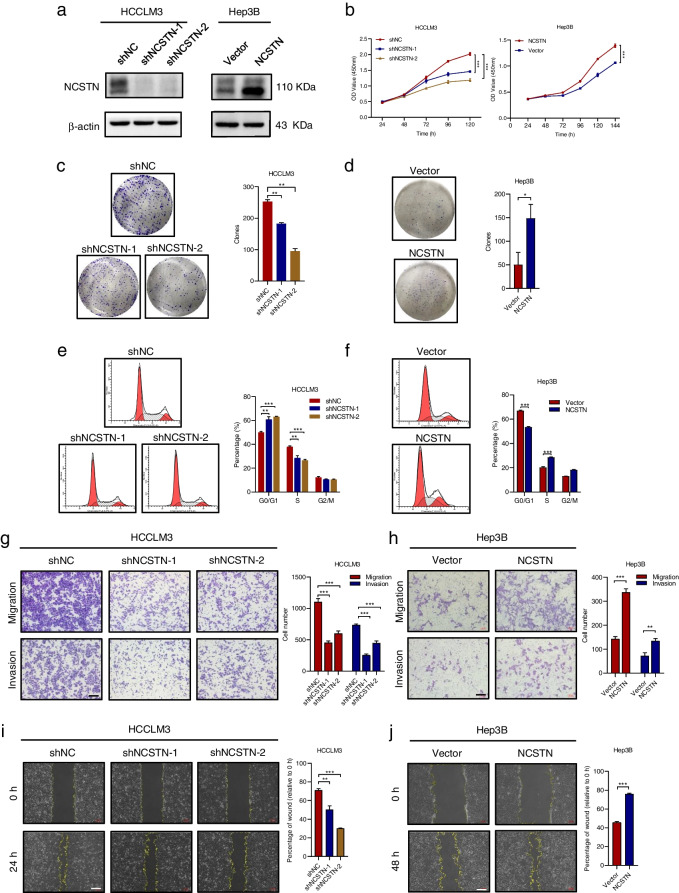
NCSTN promotes HCC cell growth and metastasis in vitro. **a** The effects of NCSTN knockdown and overexpression were examined by western blotting analysis in HCCLM3 and Hep3B cells. Loading control was assessed by β-actin. **b** CCK8 assays showed NCSTN depletion inhibited cell growth of HCCLM3 and NCSTN overexpression promoted cell growth of Hep3B. **c**,** d** Colony formation assays showed colony numbers in HCC cells with NCSTN depletion or overexpression. **e**,** f** The cell cycle assays showed that NCSTN depletion increased the G0/G1 fraction and decreased the S and G2/M fraction in HCCLM3 cells, whereas NCSTN overexpression decreased the G0/G1 fraction and increased the S and G2/M fraction in Hep3B cells. **g**,** h** The migration and invasion capacity was determined in the indicated HCC cells. Scale bar, 100 μm. **i**,** j** Wound healing assays showed the migration capacity of indicated HCC cells. Scale bar, 100 μm. HCC, hepatocellular carcinoma; CCK8, cell counting kit-8. *p < 0.05, **p < 0.01, ***p < 0.001

This correction does not change the result, interpretation, and conclusions of the study. The original article has been corrected.
