# Supplementary material for: NCSTN promotes hepatocellular carcinoma cell growth and metastasis via β-catenin activation in a Notch1/AKT dependent manner
Source: J Exp Clin Cancer Res. 2020 Jul 6;39:128. doi: 10.1186/s13046-020-01638-3 (PMC7339515; doi:10.1186/s13046-020-01638-3)
Supplement: Supplementary file 1 — Additional file 1: Table S1. Primary antibodies used in this study. Table S2. Primers used in this study. Table S3. Sequences of siRNA and shRNA used in this study. [file 13046_2020_1638_MOESM1_ESM.docx]

Table S1. Primary antibodies used in this study.

| Antigens | Antibody sources | Species | Dilution | |
| --- | --- | --- | --- | --- |
| NCSTN | Santa Cruz, sc-14369 | Goat monoclonal | 1:200 (WB), 1:100 (IHC), 1:50 (IF) |  |
| β-actin | Santa Cruz, sc-69879 | Mouse monoclonal | 1:1000 (WB) |  |
| E-cadherin | Santa Cruz, sc-8426 | Mouse monoclonal | 1:200 (WB), 1:100 (IHC), 1:50 (IF) |  |
| N-cadherin | Santa Cruz, sc-8424 | Mouse monoclonal | 1:200 (WB) |  |
| Vimentin | Santa Cruz, sc-6260 | Mouse monoclonal | 1:1000 (WB), 1:100 (IHC), 1:50 (IF) |  |
| Zeb1 | ABclonal, A1500 | Rabbit polyclonal | 1:1000 (WB), 1:1000 (IHC), 1:200 (IF) |  |
| β-catenin | CST, 8480 | Rabbit monoclonal | 1:1000 (WB), 1:1000 (IHC), 1:200 (IF) |  |
| β-tublin | Affinity, AF7011 | Mouse monoclonal | 1:1000 (WB) |  |
| Histone H3 | Affinity, AF6359 | Mouse monoclonal | 1:1000 (WB) |  |
| c-myc | ABclonal, A19032 | Rabbit monoclonal | 1:1000 (WB) |  |
| cyclin D1 | CST, 55506 | Rabbit monoclonal | 1:1000 (WB) |  |
| mmp-7 | CST, 3801 | Rabbit monoclonal | 1:1000 (WB) |  |
| Notch1 | ABclonal, A19090 | Rabbit monoclonal | 1:1000 (WB), 1:100 (IHC) |  |
| Notch2 | ABclonal, A0560 | Rabbit polyclonal | 1:1000 (WB) |  |
| Notch3 | ABclonal, A13522 | Rabbit polyclonal | 1:1000 (WB) |  |
| Notch4 | ABclonal, A8303 | Rabbit polyclonal | 1:1000 (WB) |  |
| AKT | Bioworld, BS1810 | Rabbit monoclonal | 1:1000 (WB) |  |
| p-AKT | Bioworld, BS9913M | Rabbit monoclonal | 1:1000 (WB), 1:100 (IHC) |  |
| GSK-3β | CST, 12456 | Rabbit monoclonal | 1:1000 (WB) |  |
| p- GSK-3β | CST, 5558 | Rabbit monoclonal | 1:1000 (WB) |  |
| p-β-catenin | CST, 9561 | Rabbit monoclonal | 1:1000 (WB), 1:1000 (IHC) |  |
| Ki-67 | Servicebio, GB13030-2 | Rabbit monoclonal | 1:500 (IHC) |  |

Table S2. Primers used in this study.

| Primer names | Sequences |
| --- | --- |
| NCSTN forward | GCAGCTCGAGGATGGTCTAC |
| NCSTN reverse | CCACCTGGTTCCGTACAGAC |
| CDH1 forward | AGCCCCGCCTTATGATTCTCTG |
| CDH1 reverse | TGCCCCATTCGTTCAAGTAGTCAT |
| CDH2 forward | AGTGGCAGCTGGACTTGATC |
| CDH2 reverse | CCGTGGCTGTGTTTGAAAGG |
| VIM forward | TTGAACGCAAAGTGGAATC |
| VIM reverse | AGGTCAGGCTTGGAAACA |
| ZEB1 forward | AAGTGGCGGTAGATGGTA |
| ZEB1 reverse | TTGTAGCGACTGGATTTT |
| TWIST1 forward | CTCAAGAGGTCGTGCCAATC |
| TWIST1 reverse | CCCAGTATTTTTATTTCTAAAGGTGTT |
| SNAIL1 forward | GACCCCAATCGGAAGCCTAACTAC |
| SNAIL1 reverse | AGCCTTTCCCACTGTCCTCATC |
| SNAIL2 forward | CCTCCATCTGACACCTCC |
| SNAIL2 reverse | CCCAGGCTCACATATTCC |
| FOXC1 forward | CAGCATCCGCCACAACCTCT |
| FOXC1 reverse | GCAGCCTGTCCTTCTCCTCCT |
| FOXC2 forward | GCCTAAGGACCTGGTGAAGC |
| FOXC2 reverse | TTGACGAAGCACTCGTTGAG |
| U6 forward | GCTTCGGCAGCACATATACTAAAAT |
| U6 reverse | CGCTTCACGAATTTGCGTGTCAT |
| CTNNB1 forward | GGGAGTGACTCAAGAAGTGAAG |
| CTNNB1 reverse | CAAAGCAAGCAAAGTCAGTACC |
| MYC forward | TCGGGTAGTGGAAAACCAGC |
| MYC reverse | TCCTCCTCGTCGCAGTAGAA |
| CCND1 forward | CTGATTGGACAGGCATGGGT |
| CCND1 reverse | TCTTGCCACCTCCCTTCAAC |
| Survivin forward | GAACTGGCCCTTCTTGGAGG |
| Survivin reverse | CAGCCTTCCAGCTCCTTGAA |
| MAML1 forward | GCCTCAGAGCAGCCTCTATG |
| MAML1 reverse | CATAGGCAGCTGAGACGGAG |
| HES1 forward | CACAGAAAGTCATCAAAGCC |
| HES1 reverse | CAGAATGTCCGCCTTCTC |
| CDKN1A forward | TGTCCGTCAGAACCCATGC |
| CDKN1A reverse | AAAGTCGAAGTTCCATCGCTC |
| GS forward | gaaggcctgcagagaccaat |
| GS reverse | ataggctctgtctgctccca |
| TBX3 forward | agtcgggaaggcgaatgttt |
| TBX3 reverse | gctgtccgggtgaatgtaca |
| AXIN2 forward | agcattgtctccaagcagct |
| AXIN2 reverse | ccatcaccgactggatctcg |
| BS1 forward | GACCTGATTCGGTAGGCGAC |
| BS1 reverse | GTACAGGCAGTCCTCGCTTT |
| BS2 forward | GGACTGCCTGTACTTAAAATGATCA |
| BS2 reverse | CCCACATTTTCTTGGGCATTT |
| BS3 forward | ACTCCGGTCACGTTTCAGTT |
| BS3 reverse | TTTCCTTCCTGCTTCCCACC |

Table S3. Sequences of siRNA and shRNA used in this study.

| Names | Sequences |
| --- | --- |
| shNCSTN-1 sense | 5’- CCAGUCUUCAAUUAGUGGATT-3’ |
| shNCSTN-1 anti-sense | 5’- UCCACUAAUUGAAGACUGGTT-3’ |
| shNCSTN-2 sense | 5’- CGAGGAUGGUCUACGAUAUTT-3’ |
| shNCSTN-2 anti-sense | 5’- AUAUCGUAGACCAUCCUCGTT-3’ |
| siZeb1 sense | 5’-CCUAGUCAGCCACCUUUAATT-3’ |
| siZeb1 anti-sense | 5’-UUAAAGGUGGCUGACUAGGTT-3’ |
| siβ-catenin sense | 5’-AUUACAAUCCGGUUGUGAACGUCCCTT-3’ |
| siβ-catenin anti-sense | 5’-GGGACGUUCACAACCGGAUUGUAAUTT-3’ |
