# Supplementary material for: NCSTN promotes hepatocellular carcinoma cell growth and metastasis via β-catenin activation in a Notch1/AKT dependent manner
Source: J Exp Clin Cancer Res. 2020 Jul 6;39:128. doi: 10.1186/s13046-020-01638-3 (PMC7339515; doi:10.1186/s13046-020-01638-3)
Supplement: Supplementary file 2 — Additional file 2: Supplementary figure legends [file 13046_2020_1638_MOESM2_ESM.docx]

**Supplementary figure legends**

**Figure S1.** **NCSTN promotes HCC cell growth and metastasis in vitro. a** The effects of NCSTN knockdown and overexpression were examined by western blotting analysis in SNU449 and Huh7 cells. Loading control was assessed by β-actin. **b** CCK8 assays showed NCSTN depletion inhibited cell growth of SNU449 and NCSTN overexpression promoted cell growth of Huh7. **c, d** Colony formation assays showed colony numbers in HCC cells with NCSTN depletion or overexpression. **e, f** The cell cycle assays showed that NCSTN depletion increased the G0/G1 fraction and decreased the S and G2/M fraction in SNU449 cells, whereas NCSTN overexpression decreased the G0/G1 fraction and increased the S and G2/M fraction in Huh7 cells. **g, h** The migration and invasion capacity was determined in the indicated HCC cells. Scale bar, 100 μm. **i, j** Wound healing assays showed the migration capacity of indicated HCC cells. Scale bar, 100 μm. HCC, hepatocellular carcinoma; CCK8, cell counting kit-8. *p < 0.05, **p < 0.01, ***p < 0.001.

**Figure S2. Immunohistochemical staining of metastatic foci and EMT markers. a, b** Representative images of corresponding Ki-67 staining of liver orthotopic-implanted HCC models. **c, d** Representative images of corresponding Ki-67 staining of lung metastasis models. Scale bars, 100 μm. **e** Representative images of EMT-related markers E-cadherin, Vimentin and N-cadherin in HCC tissues by immunohistochemical staining refer to Figure 4h. Scale bars, 50 μm. **f** Correlation analyses of NCSTN and CDH1, VIM as well as ZEB1 using TCGA LIHC dataset.

**Figure S3. NCSTN promoted activation of β-catenin**. **a** Correlation between NCSTN expression and CTNNB1 expression in patients from TCGA dataset. **b** The mRNA expression levels of β-catenin target genes GS, AXIN2, TBX3 and Survivin in the indicated cells. **c** Correlation analyses of NCSTN and GS, AXIN2 as well as TBX3 using TCGA LIHC dataset. **d, e** Representative images of corresponding β-catenin staining of liver orthotopic-implanted HCC models. **f, g** Representative images of corresponding β-catenin staining of lung metastasis models. Scale bars, 100 μm. *p < 0.05, **p < 0.01, ***p < 0.001.

**Figure S4. Biological effect of NCSTN is rescued by knockdown of β-catenin in indicated cells. a, b** The mRNA and protein expression levels of β-catenin target genes in the indicated Hep3B cells co-transfected with or without siβ-catenin. **c, d** Representative images showed the migration and invasion capacity was rescued in the indicated Hep3B cells co-transfected with siβ-catenin. Scale bars, 100 μm. *p < 0.05, **p < 0.01, ***p < 0.001.

**Figure S5. NCSTN regulates β-catenin through Notch/AKT/GSK-3β signaling pathway. a** The mRNA expression levels of Notch target genes MAML1, HES1, MYC and p21 in the indicated cells. **b** Co-IP assays showed NCSTN regulated the interaction between GSK-3β and β-catenin in the indicated cells. **c, d** The migration and invasion capacity was rescued in cells treated with MK-2206 2HCl. Scale bars, 100 μm. *p < 0.05, **p < 0.01, ***p < 0.001.

Figure S6. **Correlation between NCSTN and p-AKT, nuclear NOTCH1 as well as EMT markers in human HCC collections and subcutaneous xenografts. a** Representative images of p-AKT staining in human HCC collections and relevant correlation analysis. **b** Representative images of nuclear NOTCH1 staining in human HCC collections and relevant correlation analysis. **c** Representative images of p-AKT, nuclear NOTCH1 and EMT markers in subcutaneous xenografts.
