# Supplementary figures and images for: NCSTN promotes hepatocellular carcinoma cell growth and metastasis via β-catenin activation in a Notch1/AKT dependent manner
Source: J Exp Clin Cancer Res. 2020 Jul 6;39:128. doi: 10.1186/s13046-020-01638-3 (PMC7339515; doi:10.1186/s13046-020-01638-3)

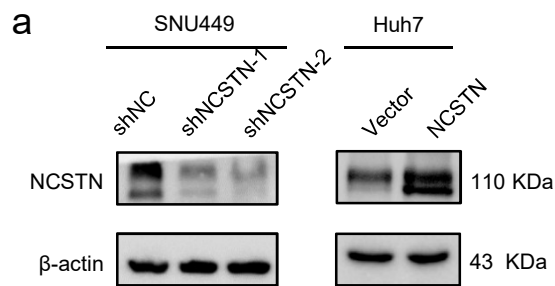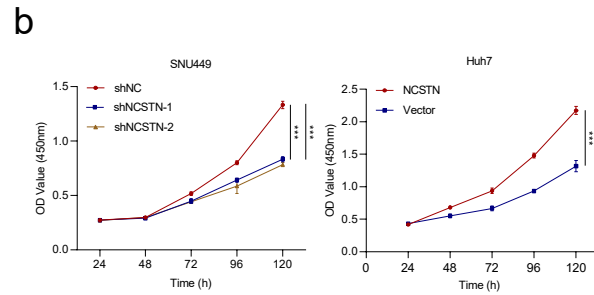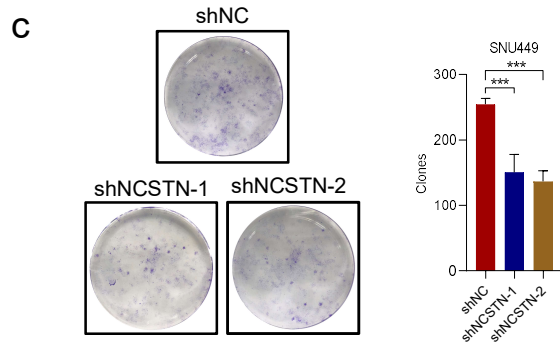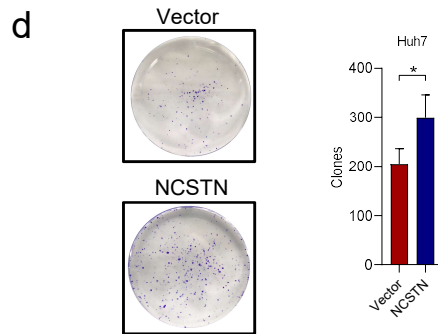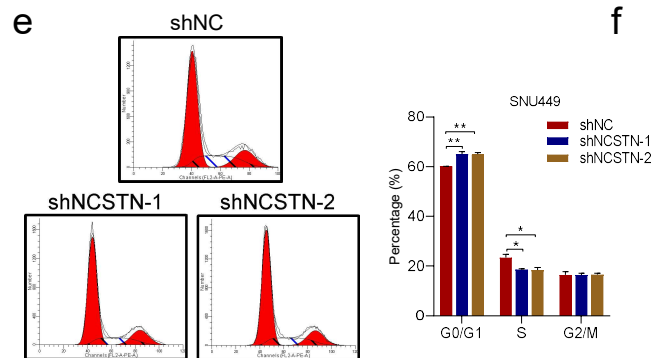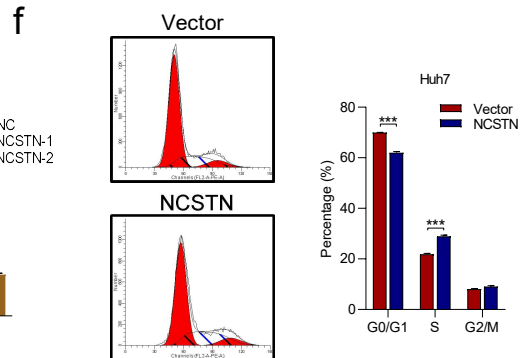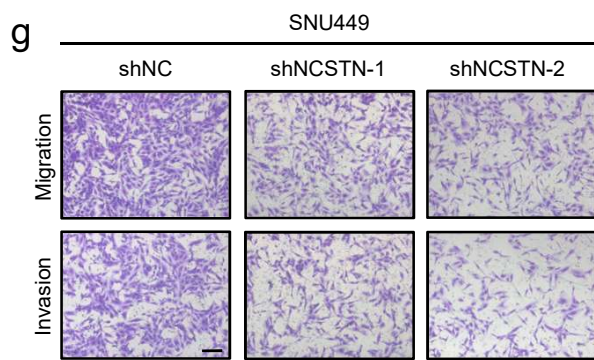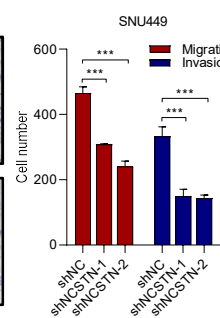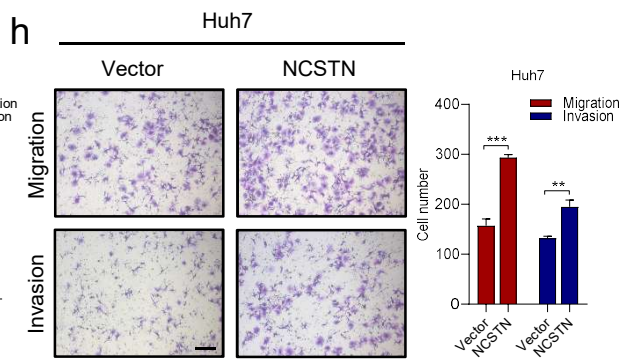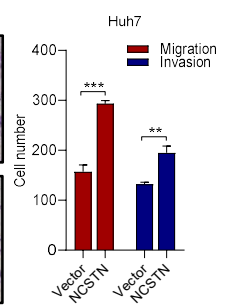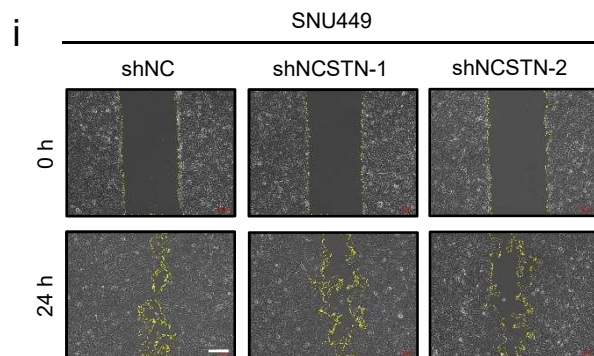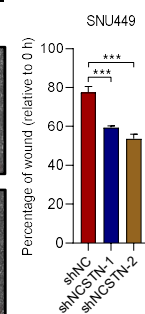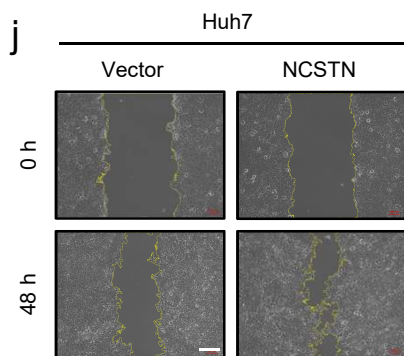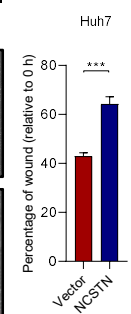

Supplement: Supplementary file 4 — Additional file 4: Figure S1. NCSTN promotes HCC cell growth and metastasis in vitro. [file 13046_2020_1638_MOESM4_ESM.pdf]

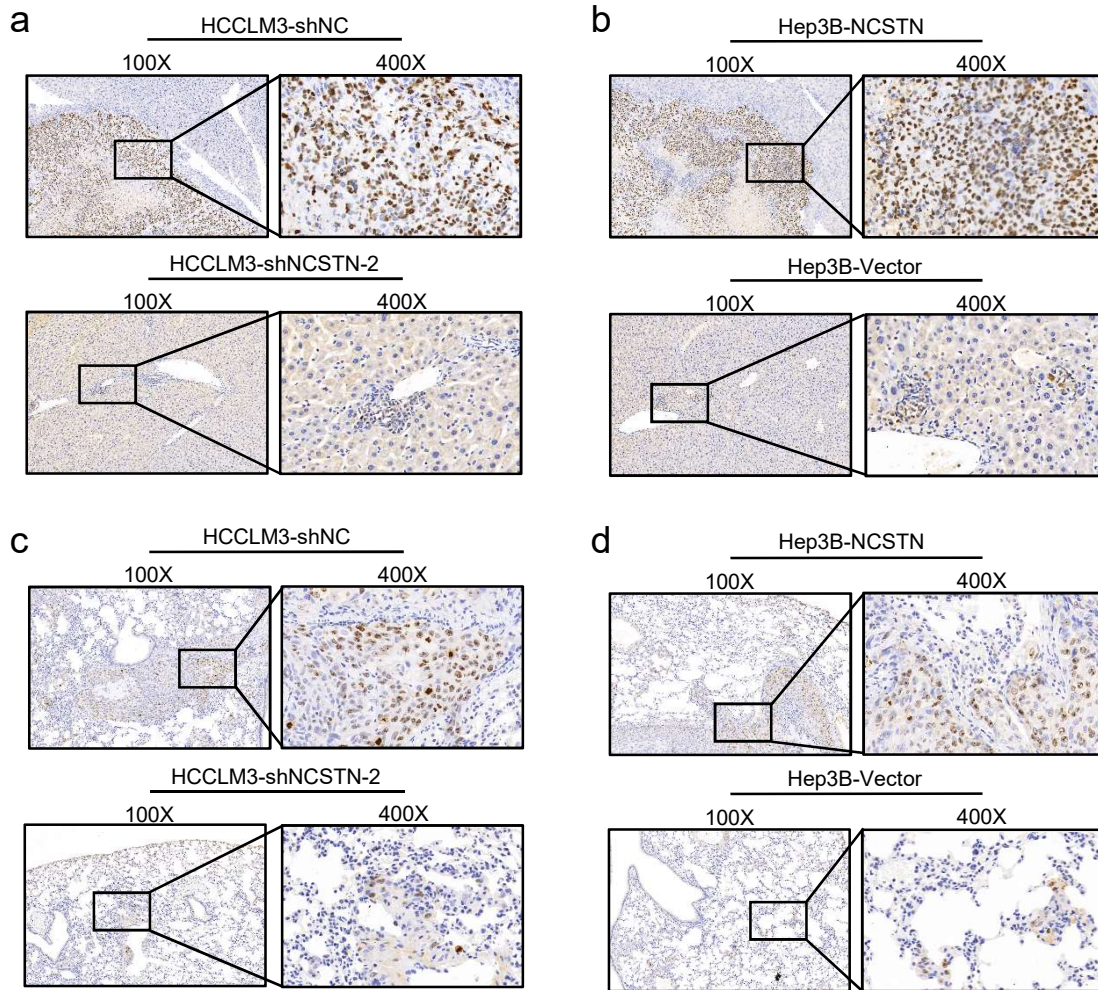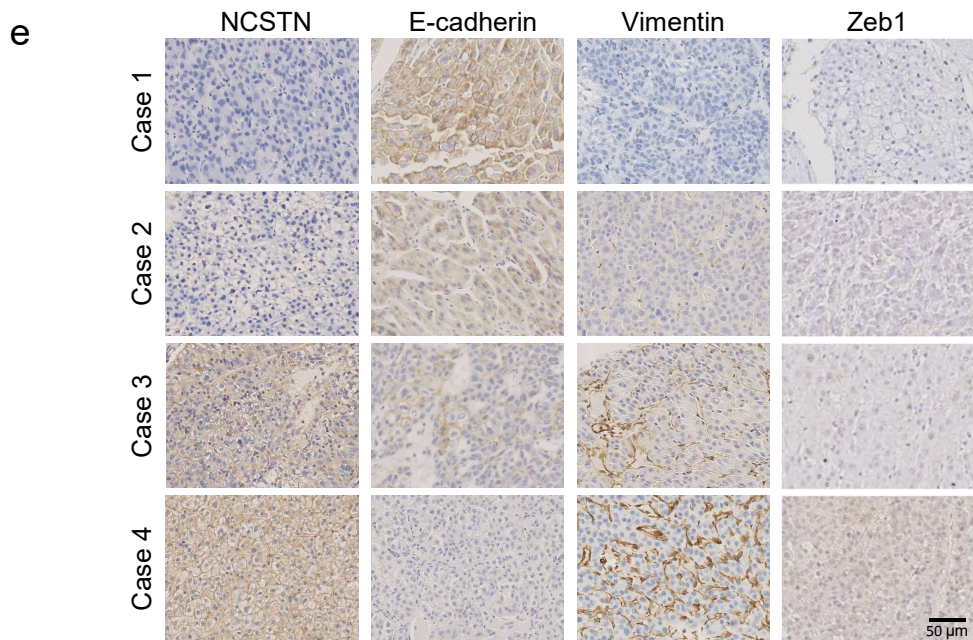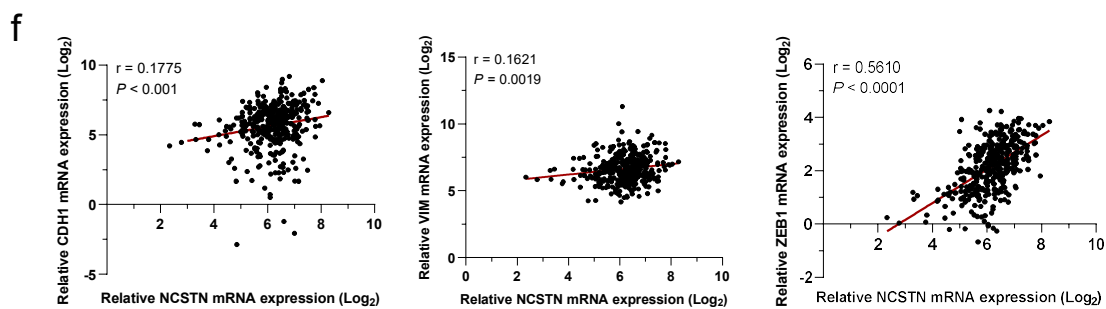

Supplement: Supplementary file 5 — Additional file 5: Figure S2. Immunohistochemical staining of metastatic foci and EMT markers. [file 13046_2020_1638_MOESM5_ESM.pdf]

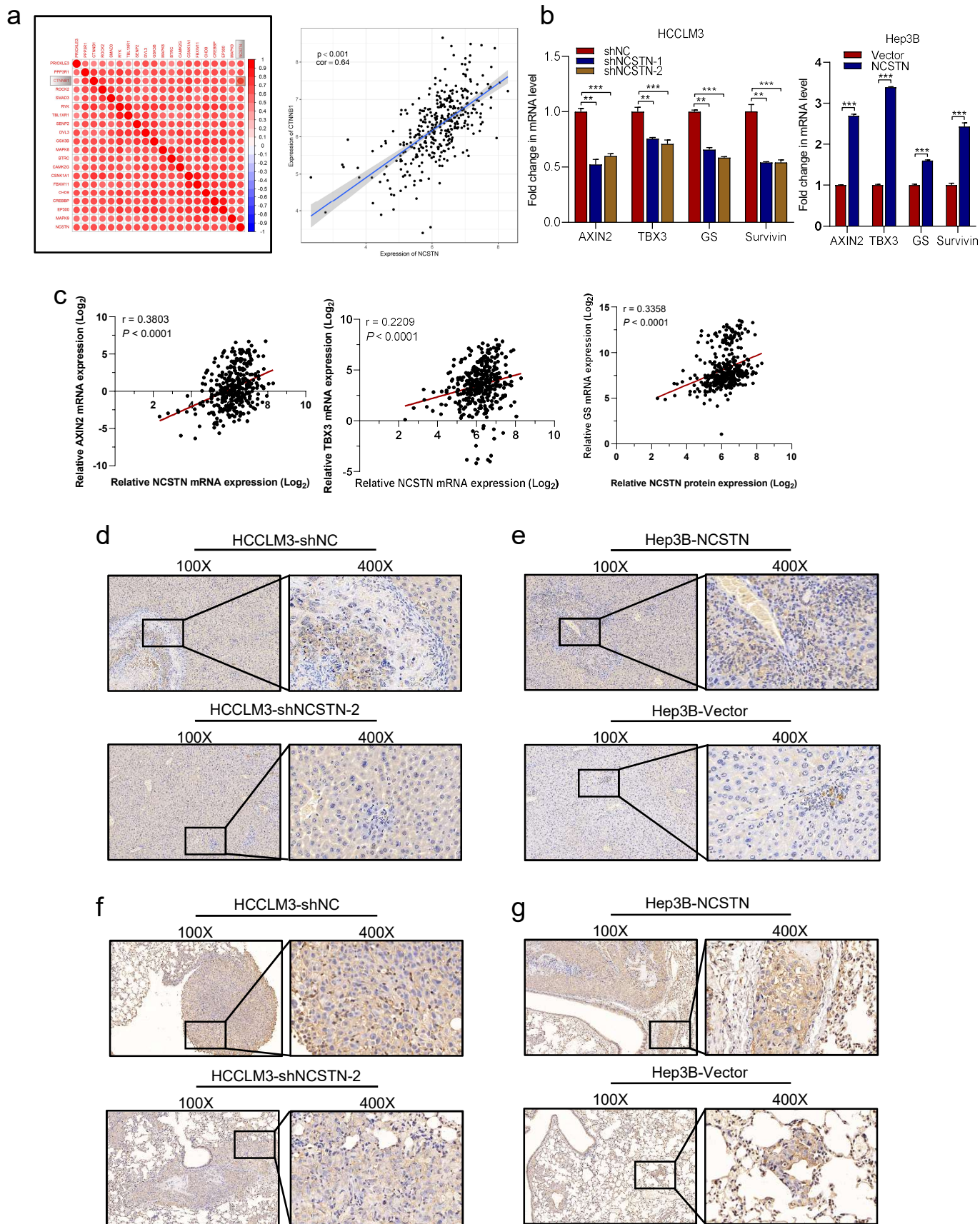

Supplement: Supplementary file 6 — Additional file 6: Figure S3. NCSTN promoted activation of β-catenin. [file 13046_2020_1638_MOESM6_ESM.pdf]

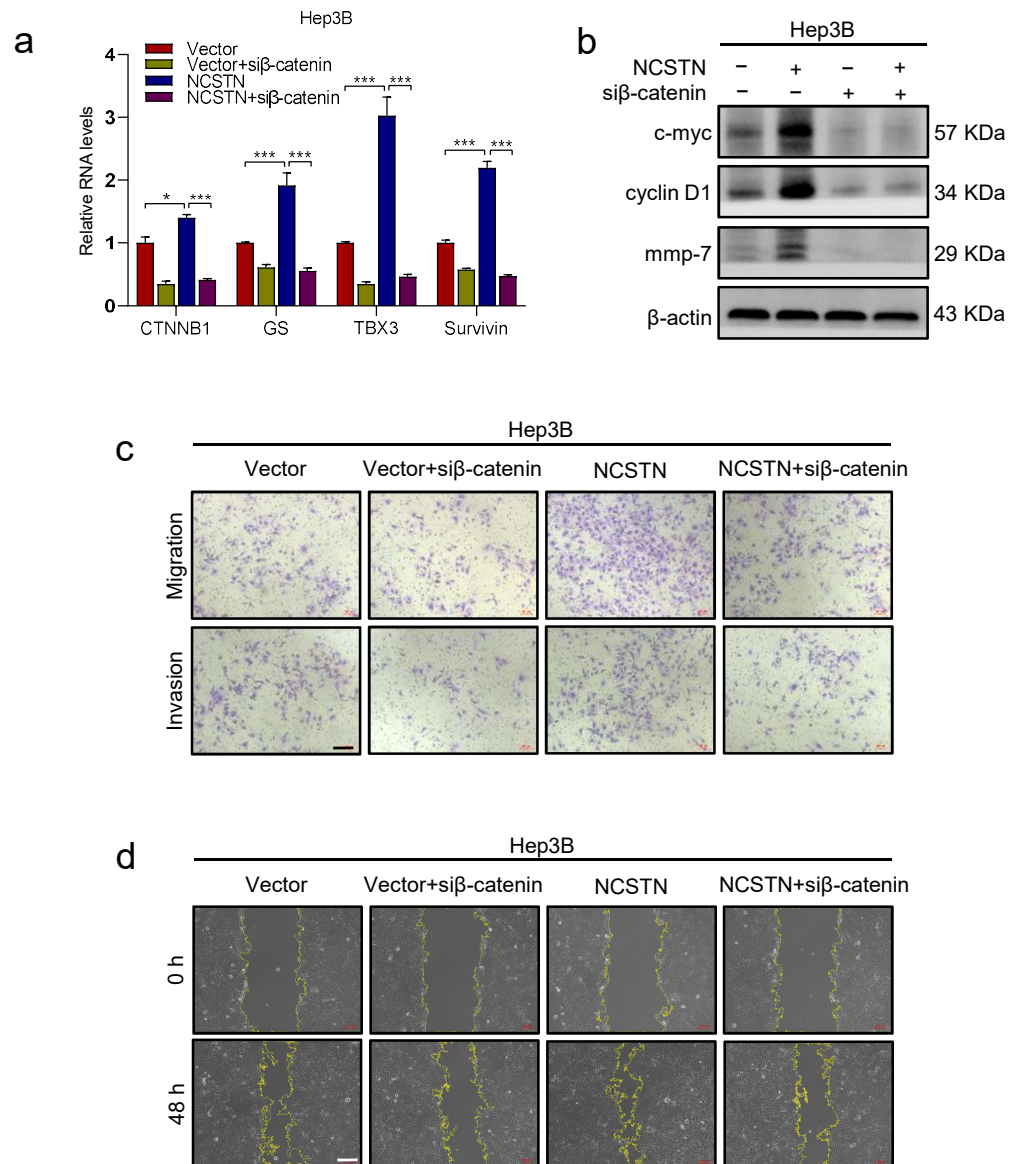

Supplement: Supplementary file 7 — Additional file 7: Figure S4. Biological effect of NCSTN is rescued by knockdown of β-catenin in indicated cells. [file 13046_2020_1638_MOESM7_ESM.pdf]

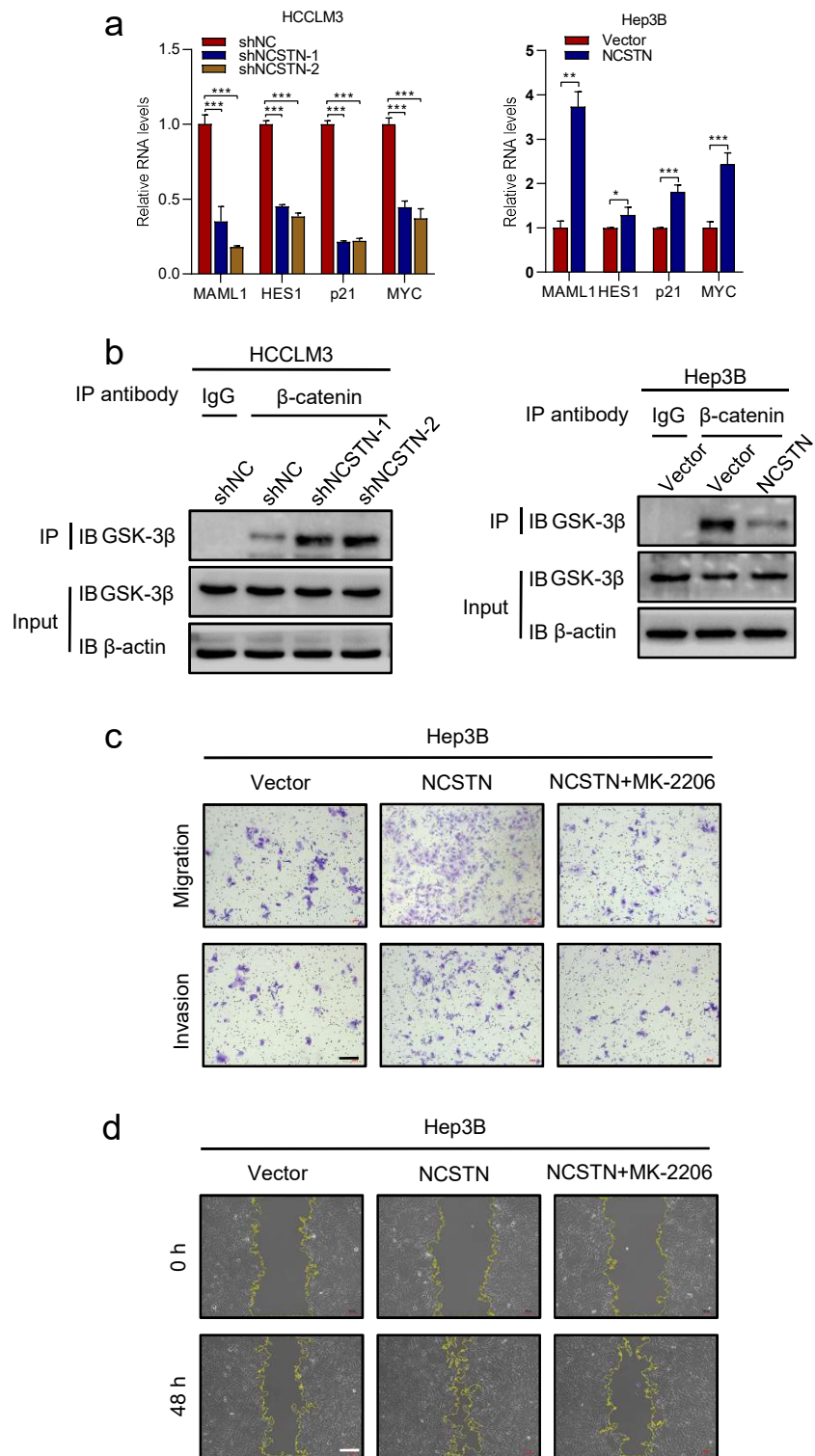

Supplement: Supplementary file 8 — Additional file 8: Figure S5. NCSTN regulates β-catenin through Notch/AKT/GSK-3β signaling pathway. [file 13046_2020_1638_MOESM8_ESM.pdf]

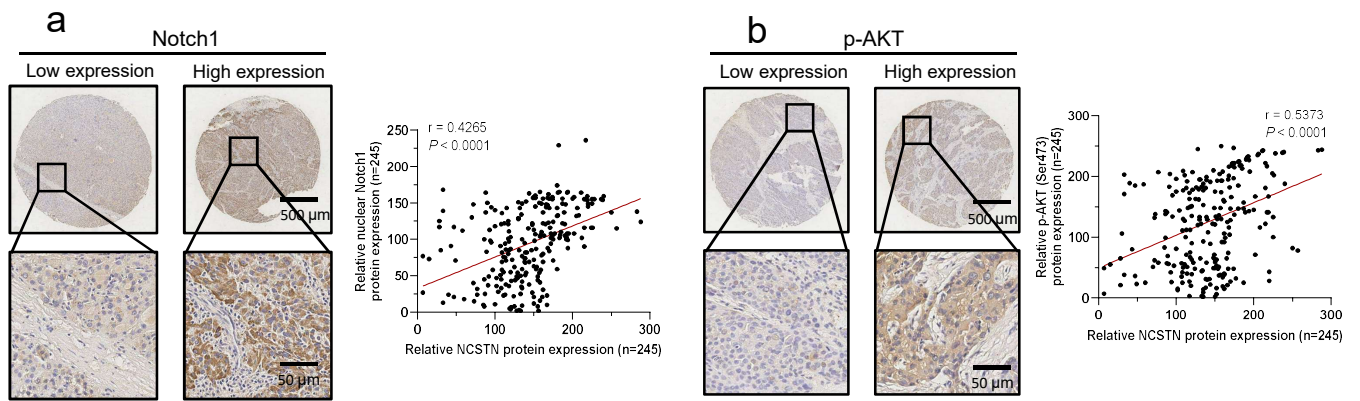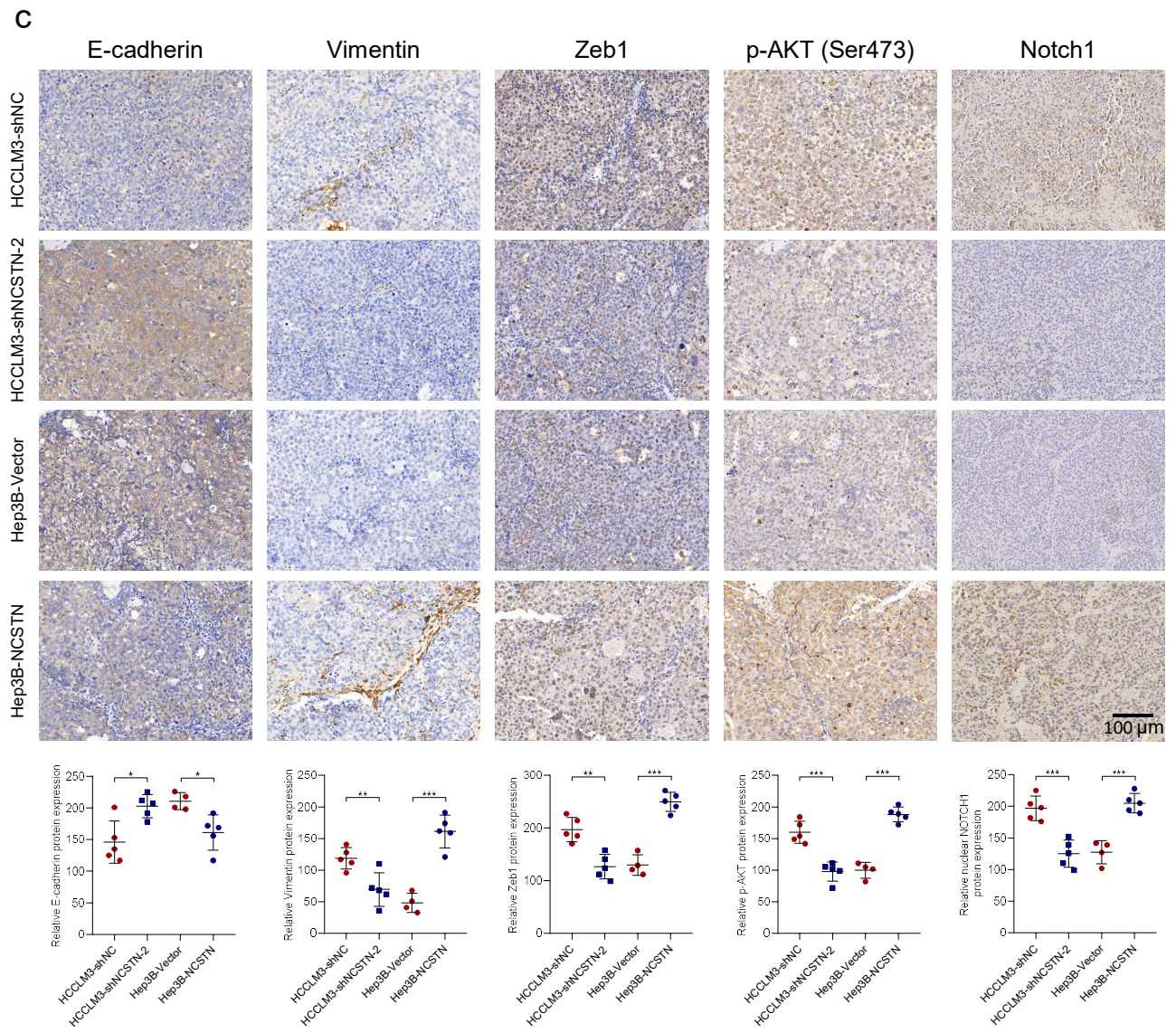

Supplement: Supplementary file 9 — Additional file 9: Figure S6. Correlation between NCSTN and p-AKT, nuclear NOTCH1 as well as EMT markers in human HCC collections and subcutaneous xenografts. [file 13046_2020_1638_MOESM9_ESM.pdf]
